# The Designer Drug αPHP Affected Cell Proliferation and Triggered Deathly Mechanisms in Murine Neural Stem/Progenitor Cells

**DOI:** 10.3390/biology12091225

**Published:** 2023-09-11

**Authors:** Elisa Roda, Fabrizio De Luca, Erica Cecilia Priori, Daniela Ratto, Silvana Pinelli, Emilia Corradini, Paola Mozzoni, Diana Poli, Giuliano Mazzini, Maria Grazia Bottone, Anna Maria Gatti, Matteo Marti, Carlo Alessandro Locatelli, Paola Rossi, Daniele Bottai

**Affiliations:** 1 Laboratory of Clinical & Experimental Toxicology, Pavia Poison Centre, National Toxicology Information Centre, Toxicology Unit, Istituti Clinici Scientifici Maugeri IRCCS, 27100 Pavia, Italycarlo.locatelli@icsmaugeri.it (C.A.L.); 2 Department of Biology and Biotechnology “L. Spallanzani”, University of Pavia, 27100 Pavia, Italy; fabrizio.deluca01@universitadipavia.it (F.D.L.); paola.rossi@unipv.it (P.R.); 3 Department of Medicine and Surgery, University of Parma, 43126 Parma, Italy; 4 INAIL Research, Department of Occupational and Environmental Medicine, Epidemiology and Hygiene, Via Fontana Candida, 1, 00078 Monte Porzio Catone, Italy; 5 Institute of Molecular Genetics—CNR (National Research Council), 27100 Pavia, Italy; 6 Department of Translational Medicine, Section of Legal Medicine, LTTA Center and University Center of Gender Medicine, University of Ferrara, 44121 Ferrara, Italy; matteo.marti@unife.it; 7 Collaborative Centre for the Italian National Early Warning System, Department of Anti-Drug Policies, Presidency of the Council of Ministers, 44121 Ferrara, Italy; 8 Department of Pharmaceutical Sciences, Section of Pharmacology and Biosciences, University of Milan, Via Balzaretti 9, 20133 Milan, Italy; daniele.bottai@unimi.it

**Keywords:** new psychoactive substances, synthetic cathinones, αPHP, neural stem/progenitor cells, CNS toxicity, cell death pathways, ultrastructural damage, mitochondrial toxin

## Abstract

**Simple Summary:**

In recent decades, the world has faced an emerging problem, which is assuming increasingly impressive dimensions: the appearance of New Psychoactive Substances, characterized by peculiar pharmacological and toxicological properties, and are exceptionally dangerous for consumers health. Among novel psychoactive substances, synthetic cathinones have recently emerged on the market and, based on their low cost and availability in smartshops and online, their use has been growing extremely, particularly among adolescents and young adults. However, the understanding of neurotoxic mechanisms induced by synthetic cathinones is still lacking, particularly on neural stem/progenitor cell cultures. In the current in vitro study, the effects of increasing αPHP concentrations, one of major MDPV derivatives, on cell viability/proliferation, morphology/ultrastructure, and cell death pathways, have been evaluated after exposure in murine neural stem/progenitor cells, using a battery of complementary techniques. We revealed several different alterations after αPHP treatment, indicating the potential harmful effects of this synthetic cathinone on young brains. Hence, the present investigation could pave the way for a broadened understanding of the synthetic cathinone toxicology needed to establish an appropriate treatment for novel psychoactive substances and the potential consequences for public health.

**Abstract:**

Increasing reports of neurological and psychiatric outcomes due to psychostimulant synthetic cathinones (SCs) have recently raised public concern. However, the understanding of neurotoxic mechanisms is still lacking, particularly for the under-investigated αPHP, one of the major MDPV derivatives. In particular, its effects on neural stem/progenitor cell cultures (NSPCs) are still unexplored. Therefore, in the current in vitro study, the effects of increasing αPHP concentrations (25–2000 μM), on cell viability/proliferation, morphology/ultrastructure, genotoxicity and cell death pathways, have been evaluated after exposure in murine NSPCs, using a battery of complementary techniques, i.e., MTT and clonogenic assay, flow cytometry, immunocytochemistry, TEM, and patch clamp. We revealed that αPHP was able to induce a dose-dependent significant decrease of the viability, proliferation and clonal capability of the NSPCs, paralleled by the resting membrane potential depolarization and apoptotic/autophagic/necroptotic pathway activation. Moreover, ultrastructural alterations were clearly observed. Overall, our current findings demonstrate that αPHP, damaging NSPCs and the morpho-functional fundamental units of adult neurogenic niches may affect neurogenesis, possibly triggering long-lasting, irreversible CNS damage. The present investigation could pave the way for a broadened understanding of SCs toxicology, needed to establish an appropriate treatment for NPS and the potential consequences for public health.

## 1. Introduction

In recent years, the entire world has faced an emerging problem which is assuming increasingly impressive dimensions. The appearance of New Psychoactive Substances (NPS), characterized by peculiar pharmacological and toxicological properties, are exceptionally dangerous for consumers health, therefore representing a pressing matter of concern for National Regulatory Agencies and International Health Regulatory Bodies. In recent decades more than 600 NPS have been identified, highlighting the exponential growth in the “legal” and illegal market of new NPSs, with a consequent increase in reports of cases of toxicity and adverse effects [1,2,3,4,5].

Hence, although the currently available data are neither exhaustive nor conclusive, the toxicity of NPSs is extremely worrying, being the cause of immediate death and/or irreversible functional alterations both at the CNS level, as well as at the systemic level and in peripheral organs. The clinical relevance found in cases of acute NPS toxidromes [1,4,5,6,7,8,9] is also aggravated by the absolute lack of knowledge regarding the medium and its long-term effects profile, including their potential for abuse, dependence and withdrawal and the possible effects of neurotoxicity being able to induce permanent brain deficits.

Synthetic cathinones (SCs), β-keto-amphetamine analogues, are one of the most prevalent designer drugs that are widely offered by the global illicit market [10,11,12]. The main mechanism underlying their pharmacological actions is dysregulation of the central monoaminergic pathways, often correlated with fatal and non-fatal intoxications [13,14,15,16].

SCs, commonly known as “legal highs” and “bath salts” including 3,4-methylenedioxypyrovalerone (MDPV), Mexedrone (3-methoxy-2-(methylamino)- 1-(4-methylphenyl)propan-1-one), αPVP (alpha-pyrrolidinopentiophenone), and αPHP (or PV7 or α-pyrrolidinohexaphenone, 1-phenyl-2-(10-pyrrolidinyl)-1-hexanone), induce psychostimulant effects similar to those triggered by cocaine, amphetamine, and MDMA [16,17].

αPHP, a less common analogue of αPVP, is a one of the pyrovalerone derivatives (-pyrrolidinophenones) known to be a SCs subgroup chemically characterized by a pyrrolidine ring and a long side chain linked to the -carbon [18,19]. The first report on αPHP identification occurred in 2014 in Japan [17,20]. αPHP has been frequently linked to polydrug abuse cases and, similarly to other SCs, provokes stimulant effects such as euphoria, sociability, intensified sensory experiences, increased energy, and reduced appetite [17,21]. Parallelly, several adverse effects including, but not limited to, agitation, restlessness, hallucinations, psychosis, seizures, tachycardia and bruxism [21,22,23,24] have been reported [13,14,25,26]. A total of 13 α-PHP-related deaths were reported to the early warning system of the European Monitoring Centre for Drugs and Drug Addiction (EMCDDA) between 2017 and 2020, with additional cases reported to the United Nations Office on Drugs and Crime (UNODC) early warning advisory system. In December 2019, the World Health Organization (WHO) recommended the control of α-PHP under Schedule II of the UNODC convention on psychotropic substances of 1971 [27].

Furthermore, several in vivo studies have been conducted to identify the neurotoxicity mechanisms underlying the SCs-triggered adverse clinical manifestations. Typical hallmarks comprise inflammation, disruption of monoaminergic neurotransmitters, alterations in thermoregulation, oxidative stress, and cytotoxicity [5,17,28,29].

The pharmacological activity of pyrovalerone derivatives has been also investigated, revealing that αPHP was able to trigger a selective and potent inhibition of dopamine and norepinephrine reuptake, showing instead negligible effects on serotonin transporters and also lacking the ability to increase neurotransmitter release [4,17,25,30,31].

Recently, in vitro studies attempted to identify the neurotoxic potential of several SCs, identifying oxidative stress, disrupting neuronal Ca^2+^ homeostasis disruption and mitochondrial dysfunction as fundamental events involved in SC-induced neurotoxicity [4,5,32].

Nonetheless, the understanding of neurotoxic mechanisms is still lacking, particularly for some SCs that have been relatively under-investigated, for example αPHP. Hence, the urgent need emerged for further in-depth studies to broaden knowledge of their pharmaco-toxicological profile and neurotoxic effects [9,33].

To fill this gap, we intended to investigate αPHP neurotoxic effects using a relevant in vitro model, i.e., neural stem/progenitor cell cultures (NSPCs) isolated from 8 weeks old C57BL/6 mice, characterized by peculiar staminal properties. In particular, NSPCs are self-renewing cells which can differentiate into multiple neural lineages, during embryo development, perinatal period and adult life, located in some restricted regions of adult mammalian brain, e.g., the subventricular zone (SVZ) and the hippocampus [34]. These cells are essential for brain development and brain physiological functions. Notably, NPSC in vitro expresses multipotentiality, i.e., the ability to give rise to the three major neural cell lineages: astrocytes, oligodendrocytes, and neurons [34,35,36,37]. In the present study, the NPSCs were collected from the SVZ, one of the neurogenic niches in the adult murine brain. It is well known that neurogenesis is the process of generation of newborn neurons from NPSCs, located in precise and limited areas, occurring beyond development during the adult mammalian life [38,39]. The central nervous system (CNS) is particularly vulnerable to NPS, and perturbation of adult neurogenesis contributes to several long-lasting irreversible impairments, including neuroplasticity alteration. Therefore, NPSCs have been recently employed as a valuable alternative model to explore neurotoxicity [39,40].

In the current in vitro study, the effects of increasing αPHP concentrations (25–2000 μM), one of major MDPV derivatives, on cell viability/proliferation, morphology/ultrastructure and cell death pathways, have been evaluated after 72 h-exposure in murine NSPCs, using a battery of complementary techniques, i.e., MTT and clonogenic assay (this latter evaluated after 8 days-exposure), flow cytometry, immunocytochemistry, TEM and patch clamp. We revealed several different alterations after αPHP treatment, indicating the potential harmful effects of this SC on young brains.

## 2. Materials and Methods

### 2.1. Cell Culturing: Dissection of the Brain and Preparation of Murine Neural Stem and Progenitor Cells (NSPCs)

The procedure was performed as previously described [41,42,43]. Briefly, brains were removed and tissues containing the SVZ were dissected out. Tissues derived from 8-week-old C57BL/6 mice were used to generate each culture. During dissection, tissues were maintained in a phosphate buffer (PB) solution (0.01 M) containing penicillin/streptomycin (100 U/mL each) (Invitrogen, San Diego, CA, USA) and glucose (0.6%) at 4 °C. We performed an enzymatic treatment using an Earl’s balanced salt solution (EBSS) (Sigma-Aldrich, St. Louis, MO, USA) containing 1 mg/mL papain (27 U/mg; Worthington DBA, Lakewood, NJ, USA), 0.2 mg/mL EDTA (Sigma-Aldrich), and 0.2 mg/mL cysteine (Sigma-Aldrich), for 45 min at 37 °C on a rocking platform. Tissues were then centrifuged at 123× *g*, the supernatant was discarded, and the pellet was re-suspended in proliferation medium (PM) [34]. Under these conditions, in 3–5 days NSPCs present in brain tissue gave rise to spheroidal structures, namely neurospheres, which were harvested, mechanically dissociated and re-plated in PM at a concentration of 10,000 cells/cm^2^.

### 2.2. Experimental Design

#### 2.2.1. Synthetic Cathinone αPHP

The synthetic cathinone αPHP, purchased from LGC Standards (LGC Standards S.r.L., Sesto San Giovanni, Milan, Italy), was kindly provided by Prof. Matteo Marti (Section of Legal Medicine and LTTA Centre, Department of Translational Medicine, University of Ferrara, 44121 Ferrara, Italy). αPHP was dissolved in phosphate-buffered saline (PBS) (Sigma Aldrich, Milan, Italy) up to 40 mM stock solution and stored at −20 °C until use.

#### 2.2.2. NSPCs Exposure to NPS: Cell Morphology, Viability, and Proliferation

To narrow down the range to be employed in the following analyses, as the first experimental step, a range of αPHP concentrations, i.e., 25, 50, 100, 200, 500, 1000 and 2000 μM, chosen based on previous literature data [5,44,45,46,47,48,49,50], was evaluated through the following tests:

*(i)*  *Phase-Contrast Microscopy: NSPCs Morphology Characterization*

After αPHP exposure, cells were counted in order to determine their proliferation capability by means of the dissociation of the neurospheres. Single cells were plated on 10 mm coverslip Cultrex^®^-coated (Tema Ricerca S.r.l., Castenaso, Italy), at a density of 15,000–20,000 cells per coverslip, left to attach for 45 min, then fixed in 4% paraformaldehyde, washed in PBS and kept at 4 °C until the following analysis.

NSPCs were observed under inverted phase contrast microscopy equipped with a 20× objective (Olympus CKX41) after αPHP exposure (25–2000 μM) for 72 h to evaluate possible cell morphology changes/alteration. Digital micrographs were shot with a camera (Olympus MagniFire digital camera), stored on a PC and processed with the Olympus Cell F software (version n. 3.1).

The analysis of cell density was performed using ImageJ software 1.54 (NIH, Bethesda, MD, USA). Specifically, four random images in each well (excluding parts near the edge of the well) were acquired at 32× magnification for each replicate at each concentration, and then converted to binary images and analyzed with the “particle analysis tool”. The cell density was calculated as reported below: number of cells/area in mm^2^.

*(ii)*  *Proliferation assay*

Growth curves were obtained from NSPC cultures between the 3rd passage (P3) and the 10th (P10). At each passage, cells were mechanically dissociated when the neurospheres reached a dimension of about 0.1 mm. Therefore, they were plated at a density of 10,000 cells/cm^2^ in a 1 cm^2^ well (48 well plate) and maintained in medium added with selected αPHP concentrations (25–2000 µM) for 72 h (3 days; this duration was chosen because NSPCs exponential growth arises between 3–5 days); then, the neurospheres were dissociated and the cells were counted. The cumulative total number of cells for each passage was calculated by multiplying the proliferation rate (viable cell harvest number/inoculum cell number) by the cumulative total number of cells of the previous passage [41]. As a good laboratory practice, an appropriate control compound of known toxic effect was included, i.e., ethanol (70 mM), being a well-known neurotoxicant able to cause detrimental effects in NSPCs [51,52].

*(iii)*  *MTT Assay: cell metabolic activity study*

The MTT assay was employed to evaluate cell viability by mitochondrial metabolism measuring the reduction of 3-(4,5-dimethyl-2-thiazolyl)-2,5-diphenyl-2H-tetrazolium (MTT) bromide by mitochondrial dehydrogenases. MTT assay was performed as previously described [41] with slight modifications. Briefly, cells (5000/well seeded in a 96-well plate) were exposed to different αPHP concentrations (25–2000 µM) for 72 h in 100 μL PM at 37 °C. Then, MTT (0.5 mg/mL, final concentration) was added to each well and kept for 3 h. Following this incubation (at 37 °C h in a humidified atmosphere, containing 5% CO_2_), the medium was carefully removed avoiding aspirating the neurospheres (which grow as floating neurospheres, but settled on the bottom of the well), and subsequently, the formazan crystals, formed by mitochondrial dehydrogenases, were dissolved in DMSO (100 μL/well) and quantified by measuring absorbance at 550 nm using a microplate reader (Reader Sunrise, Tecan, Milan, Italy) [41]. As a background value, all experiments were conducted while assessing in parallel blank samples containing αPHP and MTT in culture medium (without cells) to exclude the occurrence of non-enzymatic reduction of MTT by αPHP. Furthermore, ethanol (70 mM) was included as the positive control [51,52].

*(iv)*  *Clonal cell survival assay*

Cells were plated in a 96 well plate at a density of 150–180 cells/well in 100 µL PM containing different αPHP concentrations (25–2000 μM) and incubated for 8 days. Then, the neurospheres were counted. Specifically, only the neurospheres having a dimension bigger than 50 µm were considered, since these latter resulted from the proliferation of a single NSC, whereas smaller-sized neurospheres most likely derived from the proliferation of progenitor cells [53,54]. Plating efficiency-based (PE) calculation of survival fractions was performed as previously reported [55]. Briefly, PEs were determined by dividing the number of colonies obtained by the number of cells seeded under untreated conditions and surviving fractions were calculated by dividing the number of colonies obtained by the number of cells seeded at a given αPHP concentration multiplied for PE.

After counting clones, plating efficiency (PE) and survival fraction (SF) were calculated using the following formulas:

PE% = # of colonies formed/# of cells seeded × 100%(1)

SF= # of colonies formed after αPHP exposure/(# of cells seeded × PE)(2)

### 2.3. Immunofluorescence Reactions

Based on the obtained cell morphology, viability and proliferation data, a narrowed range of αPHP concentrations, ranging from 25 to 200 µM, was chosen to be used in the following analyses.

Control and treated NSPCs were plated and processed as previously reported for Phase Contrast microscopy evaluation, but after fixation with 4% paraformaldehyde for 10 min, cells were postfixed with 70% ethanol at −20 °C for at least 24 h and stored at −20 °C until staining. The samples were rehydrated for 15 min in PBS and then immunolabeled with selected monoclonal and polyclonal primary antibodies (summarized in Table 1) diluted in PBS. This 1 h-incubation was performed at room temperature in a dark moist chamber. Cells were then washed three times with PBS and incubated for 1 h with the proper secondary antibodies (summarized in Table 1) diluted in PBS. DNA counterstaining was therefore performed using 0.1 μg/mL of Hoechst 33258 (Sigma-Aldrich, Milan, Italy) for 6 min; then, cells were washed with PBS, and finally mounted in a drop of Mowiol (Calbiochem-Inalco S.r.l., Milan, Italy) for confocal and fluorescent microscopy. For each experimental condition, three independent experiments were carried out.

#### 2.3.1. Fluorescence Microscopy

An Olympus BX51 microscope equipped with a 100-W mercury lamp was used under the following conditions: 330–385 nm excitation filter (excf), 400 nm dichroic mirror (dm), and 420 nm barrier filter (bf) for Hoechst 33258; 450–480 nm excf, 500 nm dm, and 515 nm bf for the fluorescence of Alexa 488; 540 nm excf, 580 nm dm, and 620 nm bf for Alexa 594. Images were recorded with an Olympus MagniFire camera system (Olympus Italia S.r.l., Segrate, MI, Italy) and processed with the Olympus Cell F software (version n. 3.1).

#### 2.3.2. Confocal Fluorescence Microscopy

For confocal laser scanning microscopy, Leica TCS-SP system mounted on a Leica DMIRBE-inverted microscope was used. For fluorescence excitation, an Ar/UV laser at 364 nm was used for Hoechst 33258, an Ar/Vis laser at 488 nm was used for FITC and a He/Ne laser at 543 nm was used for Alexa 594. Spaced (0.5 μm) optical sections were recorded using a 63× oil immersion objective. The “colocalization” analysis was done considering 30 cells for sample, and three points of colocalization in at least 15 cells. Images were collected in the 1024 × 1024-pixel format, stored on a magnetic mass memory and processed by LAS-X Leica Microsystems CMS GmbH software (version n. 5.1.0).

After assessing the αPHP-induced effects as above reported, we focused on the dose of 100 µM αPHP, chosen to be used in the following additional evaluations, i.e., cytofluorimetric analysis, Patch Clamp experiments, and ultrastructural characterization by Transmission Electron Microscopy (TEM).

### 2.4. Flow Cytometry

After 72 h exposure to 100 µM αPHP, the NSPCs were detached by mild trypsinization (0.25% in PBS, with 0.05% EDTA) to obtain single-cell suspensions to be processed for flow cytometry with a Partec Cy-Flow Space system Sysmex-Partec (Milan, Italy), equipped with argon ion laser excitation at 488 nm (power 200 mW). Data were analyzed with the built-in software (Flowmax, Partec, version n. 2.70).

#### Cell Cycle Analysis and Identification of Dead Cell

A set of NSPC samples was washed in PBS, fixed in cold (−20 °C) 70% ethanol for at least 60 min and stored refrigerated (+4 °C) until all samples were recovered. Subsequently, samples were processed for cell cycle assessment by flow cytometry: fixed cells were washed in PBS to remove ethanol and re-suspended in PBS containing RNase A 100 U/mL, Nonidet P 40 0.1% (Sigma-Aldrich, Milan, Italy) and propidium iodide (PI) 50 µg/mL (Sigma-Aldrich, Milan, Italy). Cells were kept in this staining solution for at least 24 h before flow analysis.

Another set of NSPC samples were processed for the identification of dead cells. Briefly, the NSPCs were quickly washed in PBS, permeabilized in 70% ethanol for 10 min, treated with RNase A 100 U mL^−1^ and then stained at room temperature with propidium iodide (PI) 50 μg/mL (Sigma-Aldrich, Milan, Italy) 1 h before flow cytometric analysis. For both analyses, PI red fluorescence was detected with a 610 nm long-pass emission filter. At least 20,000 cells per sample were measured to obtain the distribution among the different phases of the cell cycle and to assess the percentage of dead cells normalized to control.

### 2.5. Patch Clamp Experiments

Control and 100 µM αPHP-treated NSPCs were plated in a 6-multiwell plate containing a 22 × 22 mm Cultrex^®^-coated coverslip glass (Tema Ricerca S.r.l., Castenaso, Italy) in PM. Whole-cell patch clamp was executed by using Axopatch-200B (Axon Instruments, Union City, CA, USA) patch clamp amplifier at the output cut-off frequency of 5 kHz. Data were sampled with a Digidata-1440 interface. The extracellular solution contained NaCl 140 mM, KCl 5 mM, HEPES 10 mM, glucose 10 mM, CaCl_2_ 3 mM, and MgSO_4_ 1.2 mM, and the pH was adjusted to 7.4 with NaOH. Patch pipettes were pulled from borosilicate capillaries (Hingelberg, Malsfeld, Germany) and had a mean resistance of 7 MΩ. The intrapipette solution contained potassium gluconate 126 mM, NaCl 4 mM, glucose 15 mM, HEPES 5 mM, MgSO_4_ 1 mM, BAPTA 0.1 mM, CaCl_2_ 0.02 mM, ATP 3 mM, and GTP 0.1 mM, osmolarity 280 OSM, and pH was adjusted to 7.2 with KOH. TEA 20 mM was used in patch-clamp experiments. All reagents were purchased from Sigma-Aldrich (Milan, Italy).

### 2.6. Transmission Electron Microscopy (TEM): UA and LC Staining

Control and 100 µM αPHP-treated cells were harvested by mild trypsinization (0.25% trypsin in PBS containing 0.05% EDTA) and centrifuged to obtain a visible pellet. This was immediately fixed in Karnovsky solution (4% formaldehyde, 5% glutaraldehyde) for 90 min at RT and washed with PBS (pH 7.2). Then, the pellet was embedded in agar, post-fixed in 1% OsO_4_ (Sigma Chemical Co., St. Louis, MO, USA) for 90 min at RT and dehydrated by increasing concentration of alcohol/acetone. Finally, the pellets were washed with propylene oxide and embedded in epoxy resin (Durcupan ACM, Sigma Aldrich). After resin polymerization, samples were cut at 0.5 μm thickness and stained with methylene blue and safranin to morphologically select the field of interest. Ultrathin sections were collected on a 300-mesh copper grid and stained with uranyl acetate and lead citrate. The plates, after being developed, were examined under a transmission electron microscope (Philips EM 208S), then computerized through an Epson Perfection 4990 photo scanner at a resolution of 800 dpi and then processed using the Epson Scan software (version n. 3.771).

### 2.7. Statistical Analysis

Data are presented as the mean ± SEM. The Anderson-Darling, D’Agostino & Pearson, Shapiro–Wilk and Kolmogorov–Smirnov Tests were used to establish and confirm the normality of parameters. Then, data were analysed to verify statistically significant differences. For data passed the normality test, the analysis was conducted using one-way ANOVA followed by Bonferroni’s post hoc test for multiple comparisons. Diversely, for non-normally distributed results, the analysis was performed employing the Kruskal–Wallis test followed by Dunn’s test. The differences were considered statistically significant for *p* < 0.05 (*), *p* < 0.01 (**) and *p* < 0.001 (***). For multiple comparison, the statistically significant differences were considered as follows: * compared to ctrl; Φ compared to vehicle; Ψ compared to 25 µM αPHP; # compared to 50 µM αPHP, § compared to 100 µM αPHP, Ω compared to 200 µM αPHP, γ compared to 500 µM αPHP, Ʃ compared to 1000 µM αPHP, Δ compared to 2000 µM αPHP. All statistical analyses were performed by using GraphPad Prism 8.0 (GraphPad Software Inc., San Diego, CA, USA).

## 3. Results

In the current study, murine NPSCs collected from the subventricular zone (SVZ), one of the largest germinal regions in the adult mammalian brain, which extends along the lateral wall of the telencephalic ventricles, were chosen as the in vitro model with the aim of investigating whether αPHP affects their peculiar characteristics (e.g., metabolism, proliferation, key molecules of cell death, and ultrastructure). Our final goal was to achieve a clear understanding of which mechanisms play a key role, also bearing in mind that our in vitro model, derived from 8-week-old C57BL/6 mice, could mimic the human exposure during puberty (Figure S1).

A wide αPHP concentration range (25 to 2000 µM) to be tested on NSPCs was chosen based on previous in vitro findings on SCs [5,44,45,46,47,48,49,50].

Based on the morphological evaluation by phase contrast microscopy, together with viability/proliferation data obtained by the MTT assay and clonogenic assay findings, the concentrations ranging from 25 to 200 µM αPHP were selected and employed for the following immunofluorescence evaluations. Then, the αPHP concentration of 100 µM, leading to a mild reduction (about 35%, measured by MTT assay) in the number of living/proliferation cells, was selected as the suitable sub-toxic dose to be employed for cytofluorimetric, electrophysiological and ultrastructural investigations.

### 3.1. αPHP Alters NSPCs Proliferation

**Figure 1:**
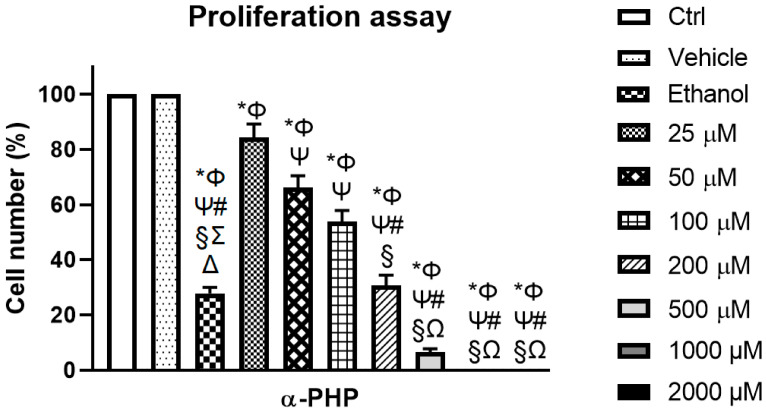
Proliferation assay showing the effect of αPHP on cell growth after 72 h-exposure. Histograms showing the NSPCs cell number (%) in control, vehicle, ethanol and differently αPHP-exposed NSPCs, i.e., 25 µM, 50 µM, 100 µM, 200 µM, 500 µM, 1000 µM and 2000 µM. Statistically significant data: * compared to ctrl; Φ compared to vehicle; Ψ compared to 25 µM αPHP; # compared to 50 µM αPHP, § compared to 100 µM αPHP, Ω compared to 200 µM αPHP, Ʃ compared to 1000 µM αPHP, Δ compared to 2000 µM αPHP.

The effects of αPHP on cell proliferation were evaluated by measuring the cell growth after 72 h-exposure to selected concentrations. Figure 1 depicts experimental results, demonstrating that already at 50 μM the treatment with αPHP induced a significant reduction (about 34%) of NSPCs proliferating capability, further exacerbating at the dose of 100 μM (cell proliferating capability decline about 46%). A further dose-dependent worsening (about 69%) was assessed at the higher concentration, i.e., 200 μM. This lessening further aggravating at the highest tested doses (about 90–100% for 500–2000 μM). In these latter experimental conditions, an almost complete cell death phenomenon was detected. The positive control (70 mM Ethanol) induced an extremely significant reduction of NSPCs proliferating capability (about 70%) (Table S1).

### 3.2. αPHP Affects Cell Density and Morphology

**Figure 2:**
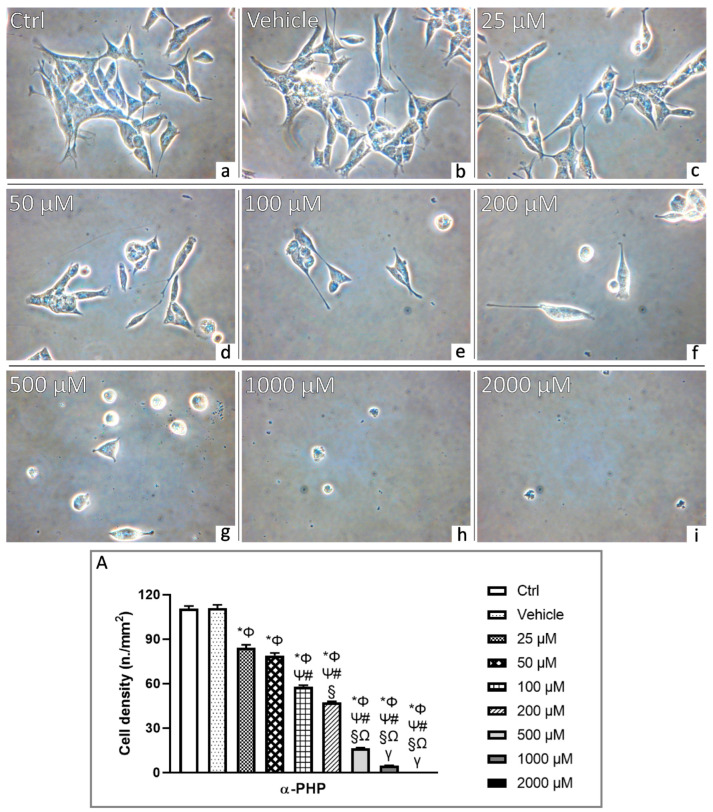
Phase-contrast microscopy of control (**a**), vehicle (**b**) and differently αPHP-exposed NSPCs (**c**–**i**) (i.e., 25 µM (**c**), 50 µM (**d**), 100 µM (**e**), 200 µM (**f**), 500 µM (**g**), 1000 µM (**h**) and 2000 µM (**i**), respectively). Histograms illustrating the quantitative cell density analysis (**A**). Statistically significant data: * compared to ctrl; Φ compared to vehicle; Ψ compared to 25 µM αPHP; # compared to 50 µM αPHP, § compared to 100 µM αPHP, Ω compared to 200 µM αPHP, γ compared to 500 µM αPHP. Magnification: 40×.

After 72 h-exposure to αPHP, NSPCs were plated on 1 cm^2^ round coverslip and examined. In detail, the phase-contrast microscopy evaluation revealed the absence of morphological changes after exposure to the lowest αPHP tested concentration, i.e., 25 µM, even though a significant decrease of cell density (about 24%) was measured. At the concentration of 50 µM αPHP, a slight further decrease in cell density (about 29%) was assessed, paralleled by an evident morphological alteration of cells, with a manifest enhancement of round-shaped cells (about 35%), often characterized by the presence of cytoplasmic blebs (Figure 2). The cell density reduction, accompanied by cell structural modifications, worsened dose-dependently, appearing more marked at higher αPHP doses, i.e., 100 µM and 200 µM, when a further cell density reduction arose (48% and 57%, respectively) (Figure 2). Notably, at 500 µM only few cells were observed (cell density decrease about 85%), showing striking signs of degeneration. Lastly, at the highest αPHP tested concentrations, i.e., 1000 and 2000 µM, only cell debris was detected, proving the occurrence of a widespread extensive cell death (Figure 2, Table S2).

Any significant alteration of cell density and morphology was determined when comparing control and vehicle-exposed cells.

### 3.3. αPHP Affects Cell Metabolism

SCs were previously reported to decrease mitochondrial respiration and MTT reduction assay has been used as a reliable tool for the determination of the cytotoxic profile of SCs [44,58,59].

**Figure 3:**
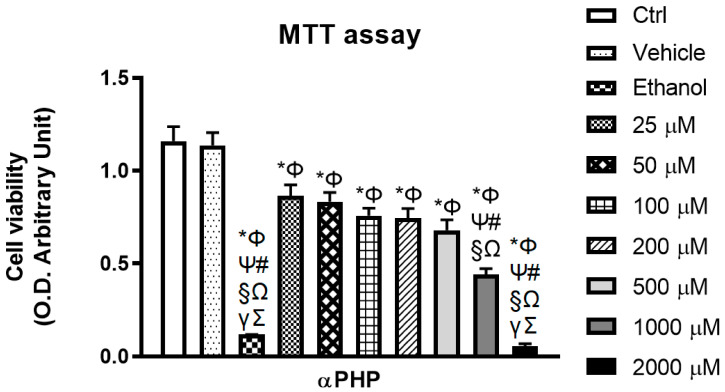
Effects of αPHP on cell viability/metabolism. Histograms showing the NSPCs viability in control, vehicle, ethanol and differently αPHP-exposed NSPCs, i.e., 25 µM, 50 µM, 100 µM, 200 µM, 500 µM, 1000 µM and 2000 µM. Statistically significant data: * compared to ctrl; Φ compared to vehicle; Ψ compared to 25 µM αPHP; # compared to 50 µM αPHP, § compared to 100 µM αPHP, Ω compared to 200 µM αPHP, γ compared to 500 µM αPHP, Ʃ compared to 1000 µM αPHP.

Currently, MTT data revealed the occurrence of a slight αPHP-induced toxic effect testing the lowest doses, with a measured viability of about 72–75% at 25 and 50 μM. Starting from the concentration of 100 μM, a more evident dose-dependent cytotoxicity was assessed, with a more marked effect at 200 μM αPHP, further exacerbated at 500 μM αPHP, which reduced the NSPCs metabolism, causing an extremely significant decrease (about 41%) in the number of living cells (Figure 3). A worsened reduction of cell viability was further detected at the highest concentrations, i.e., 1000 and 2000 μM, characterized by an extensive cell death (cell viability about 38% and 5%, respectively) (Figure 3). Concerning the 100 μM αPHP dose, able to trigger a mild reduction (about 35%) in the number of living/proliferation cells, it was selected as the suitable dose to be employed for the subsequent analyses. Positive control (70 mM Ethanol) induced an extremely significant reduction of cell viability (cell death about 89%) (Table S3).

### 3.4. αPHP Modified NSCs Clonogenic Capability

**Figure 4:**
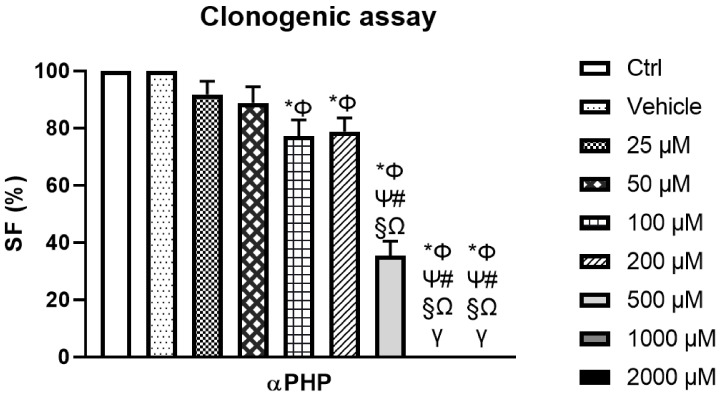
Clonogenic cell survival assay showing the dose–response effect of αPHP after 8 days-exposure. Histogram showing the NSPCs surviving fraction (SF%) in control, vehicle and differently αPHP-exposed NSPCs, i.e., 25 µM, 50 µM, 100 µM, 200 µM, 500 µM, 1000 µM and 2000 µM. Statistically significant data: * compared to ctrl NSPCs; Φ compared to vehicle NSPCs; Ψ compared to 25 µM αPHP; # compared to 50 µM αPHP, § compared to 100 µM αPHP, Ω compared to 200 µM αPHP, γ compared to 500 µM αPHP.

For the clonal analysis, the limited number of plated cells (150 per well) was crucial to avoid the formation of chimera spheres (formed by the aggregation of two or more cells), allowing to seek the assessment of αPHP-induced modification in self-renewal cells capability. Figure 4 illustrates a typical result of the eight-day clonogenic assay. We found a significant decrease in the clonogenic capability of NSCs (SF about 23%) starting from the concentration of 100 μM αPHP, further worsening at 500 μM αPHP (SF about 65%). Notably, at the highest concentrations, i.e., 1000 and 2000 μM αPHP, no NSCs clone was detected (Table S4).

### 3.5. αPHP Altered Cytoplasmic Structures and Cell Death Pathways

Based on the above reported data, a narrowed range of concentrations (25–200 μM) was tested with the aim at evaluating immunocytochemically the possible αPHP-induced cellular alterations. Specifically, the investigation focused on the apoptotic pathway and NSPCs cytoplasmic structures, i.e., cytoskeleton, mitochondria and lysosomes.

**Figure 5:**
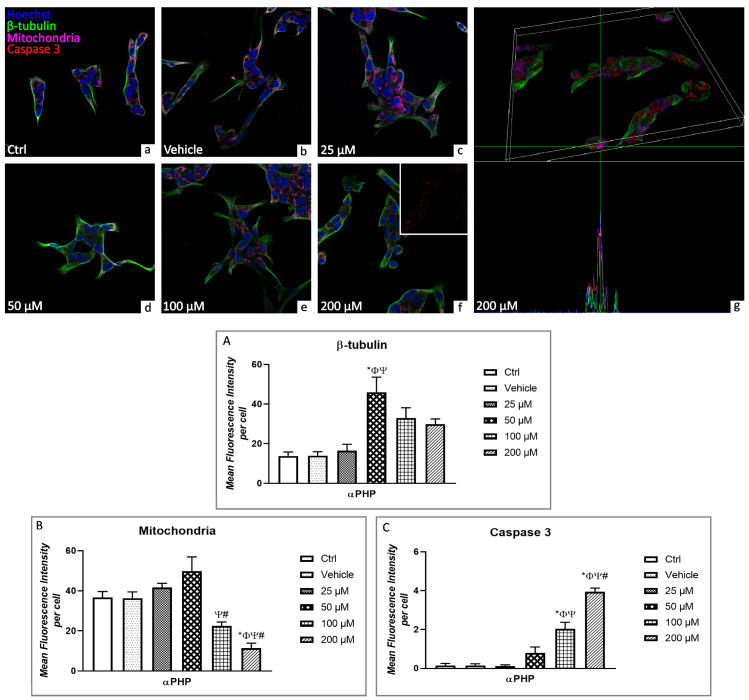
Confocal fluorescence microscopy: triple immunocytochemical detection of β-tubulin (green signal), mitochondria (magenta signal) and caspase 3 (red signal) in control (**a**), vehicle (**b**) and differently αPHP-exposed NSPCs, i.e., 25 µM (**c**), 50 µM (**d**), 100 µM (**e**) and 200 µM (**f**,**g**). DNA counterstaining with Hoechst 33258 (blue fluorescence). Lateral view of 3D 200 µM αPHP-exposed NSPCs (**g**) displaying colocalization of mitochondria (magenta fluorescence) and caspase 3 (red fluorescence). Histograms showing the quantitative assessment of β-tubulin (**A**), mitochondria (**B**) and caspase 3 (**C**) mean fluorescence intensity per cell. Statistically significant data: * compared to ctrl; Φ compared to vehicle; Ψ compared to 25 µM αPHP; # compared to 50 µM αPHP. Magnification: 60×.

Firstly, concerning the assessment of the potential activation of cell death pathway, a triple immunofluorescence staining (Figure 5) was performed to identify β-tubulin (green signal), mitochondria (magenta fluorescence) and caspase3 (red fluorescence), as specific markers. Vimentin was also investigated. In particular, Caspase-3 is generally recognized for its activated proteolytic role in the execution of apoptosis in cells responding to specific extrinsic or intrinsic stimuli [60,61].

Confocal microscopy showed mitochondria and β-tubulin labelling homogeneously distributed in the cytoplasm, with the latter predominantly localized in the peripheral region, close to the cell membrane (Figure 5). Furthermore, a lack of colocalization with cytoplasmic caspase3 (red fluorescence) was revealed investigating mitochondria and β-tubulin in control and vehicle groups (Figure 5). Notably, cell morphology changed in a dose-dependent manner, with a progressive increase of round-shaped cells (Figure 5).

The cytoskeleton reorganization was perceptible at 50 µM αPHP, when β-tubulin aggregates were clearly observed in the marginal zone of NSPCs (Figure 5).

Moreover, homogeneous mitochondria agglomerates were clearly detectable in control, vehicle, 25µM and 50µM exposed cells. However, after 100µM- αPHP exposure mitochondrial network disaggregated, and notably, the overlapping of mitochondria and caspase3 immunofluorescence signals were detected at the highest αPHP dose (i.e., 200 µM) (Figure 5).

The quantitative evaluation revealed that β-tubulin expression levels were similar comparing control, vehicle and 25 µM αPHP-treated cells. However, a significant increase was observed at 50 µM, notably followed by a slight decrease at the highest tested αPHP doses. Nonetheless, these latter β-tubulin expression values still remained slightly higher compared to those measured in the control, vehicle and 25 µM αPHP-treated NSPCs (Figure 5).

Similarly to the above-reported β-tubulin expression trend, any significant difference was measured in mitochondria immunofluorescent signal comparing control, vehicle and 25 µM αPHP-treated cells, while a slight increase of immunopositive cell OD was determined at 50 µM αPHP. Interestingly, at the highest tested doses, namely 100 and 200 µM, a significant lessening of mitochondria immunopositive cell OD was determined (Figure 5, Table S5).

**Figure 6:**
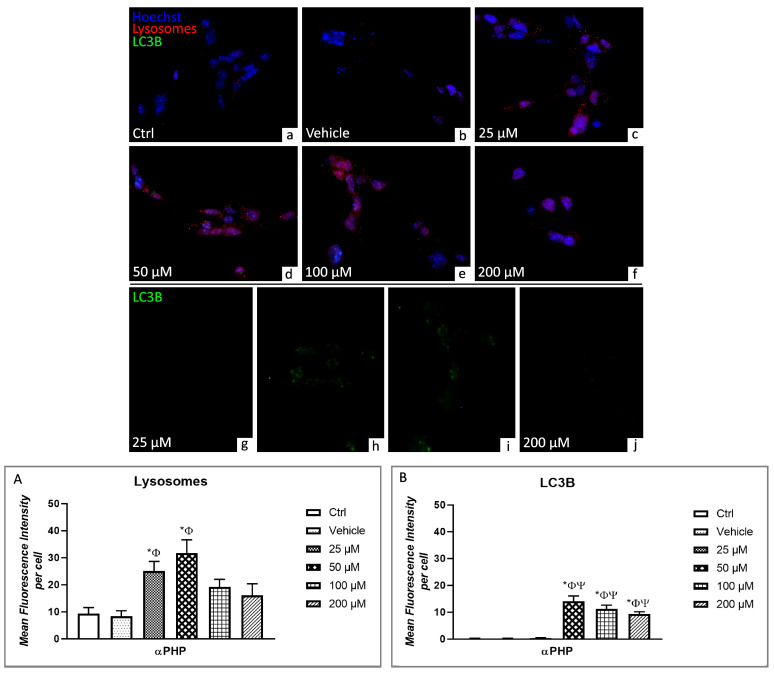
Double immunocytochemical detection of lysosomes (red signal) and LC3B (green signal) by fluorescence microscopy in control (**a**), vehicle (**b**) and differently αPHP-treated NSPCs, i.e., 25 µM (**c**,**g**), 50 µM (**d**,**h**), 100 µM (**e**,**i**) and 200 µM (**f**,**j**). DNA counterstaining with Hoechst 33258 (blue fluorescence). Histograms illustrating the quantitative assessment of lysosomes (**A**) and LC3B (**B**) mean fluorescence intensity per cell. Statistically significant data: * compared to ctrl; Φ compared to vehicle; Ψ compared to 25 µM αPHP. Magnification: 40× (**a**–**f**); 60× (**g**–**j**).

**Figure 7:**
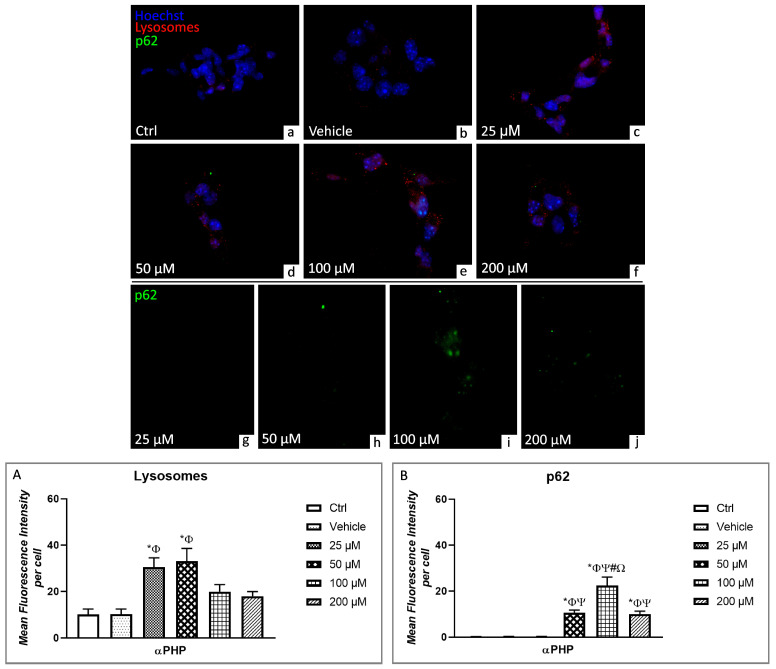
Double immunocytochemical detection of lysosomes (red signal) and p62 (green signal) by fluorescence microscopy in control (**a**), vehicle (**b**) and differently αPHP-exposed NSPCs, i.e., 25 µM (**c**,**g**), 50 µM (**d**,**h**), 100 µM (**e**,i) and 200 µM (**f**,**j**). DNA counterstaining with Hoechst 33258 (blue fluorescence). Histograms showing the quantitative evaluation of lysosomes (**A**) and LC3B (**B**) mean fluorescence intensity per cell. Statistically significant data: * compared to ctrl; Φ compared to vehicle; Ψ compared to 25 µM αPHP, # compared to 50 µM αPHP, Ω compared to 200 µM αPHP. Magnification: 40× (**a**–**f**); 60× (**g**–**j**).

Parallelly, fluorescence microscopy revealed the presence of immunolabelled lysosomes (red fluorescence) regularly distributed in the cytoplasm (Figure 6 and Figure 7). However, the presence of lysosomal vesicle agglomerates was discernible already at 25µM αPHP, with a further increase at 50µM, possibly indicating an enhanced development of autolysosomes, which could be subsequently eliminated by exocytosis, as supported by the observed dose-dependent decrease in lysosome labelling at the highest αPHP dose (i.e., 100 and 200 µM) (Figure 6 and Figure 7).

Regarding the quantitative evaluation of lysosomal alterations, any significant difference in immunopositive cell OD was determined by comparing control and vehicle NSPCs. However, a significant increase was detected when measuring immunopositive cell OD at 25 and 50 µM αPHP. Conversely, a slight reduction was highlighted at increasing doses, i.e., 100 and 200 µM. Nevertheless, these latter lysosomal immunopositive OD values still persisted slightly higher compared to those measured in Control and vehicle NSPCs (Figure 6 and Figure 7, Tables S6 and S7).

Concerning deathly pathways, the potential αPHP-induced alterations were further investigated by assessing specific markers, namely AIF, BAX, LC3B and p62.

**Figure 8:**
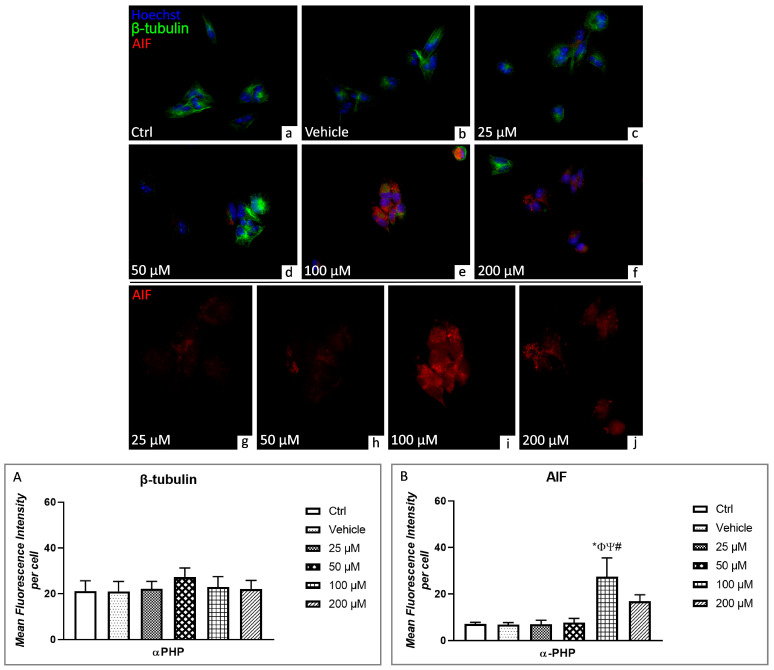
Double immunocytochemical detection of β-tubulin (green signal) and AIF (red signal) by fluorescence microscopy in control (**a**), vehicle (**b**) and differently αPHP-treated NSPCs, i.e., 25 µM (**c**,**g**), 50 µM (**d**,**h**), 100 µM (**e**,**i**) and 200 µM (**f**,**j**). DNA counterstaining with Hoechst 33258 (blue fluorescence). Histograms illustrating the quantitative assessment of β-tubulin (**A**) and AIF (**B**) mean fluorescence intensity per cell. Statistically significant data: * compared to ctrl; Φ compared to vehicle; Ψ compared to 25 µM αPHP; # compared to 50 µM αPHP. Magnification: 40× (**a**–**f**); 60× (**g**–**j**).

Apoptosis Inducing Factor (AIF) is a mitochondrion-localized flavoprotein with NADH oxidase activity, encoded by a nuclear gene, which is pivotally involved in apoptotic cell death [62,63,64]. The performed double immunofluorescence reaction for AIF (red fluorescence) and β-tubulin (green fluorescence) highlighted the lack of colocalization in control and vehicle cells (Figure 8). However, already at 25 µM αPHP, the red AIF immunofluorescence signal was discernible in the cytoplasm of several NSPCs, further increasing at 50 µM; at this latter dose, αPHP-treated cells also showed the previously described β-tubulin aggregates in the marginal zone, close to the cell membrane (Figure 8). At the highest doses (i.e., 100 and 200 µM αPHP), AIF immunolabelling was homogeneously distributed throughout the whole cytoplasm, showing to overlap with β-tubulin immunofluorescent signal in several areas, typically characterized by abundant yellow spots. This colocalization phenomenon was particularly evident after exposure to 100 µM αPHP (Figure 8, Table S8).

Regarding the quantitative evaluation of AIF immunofluorescent signal, any significant difference was assessed comparing control, vehicle, 25 µM- and 50 µM-treated cells. Interestingly, an extremely significant increase in immunopositive cell OD was measured in NSPCs exposed to 100 µM αPHP. Conversely, at the higher dose of 200 µM αPHP, a slight decrease was determined, even though this value still remained faintly higher compared to those measured in all other experimental conditions (Figure 8).

**Figure 9:**
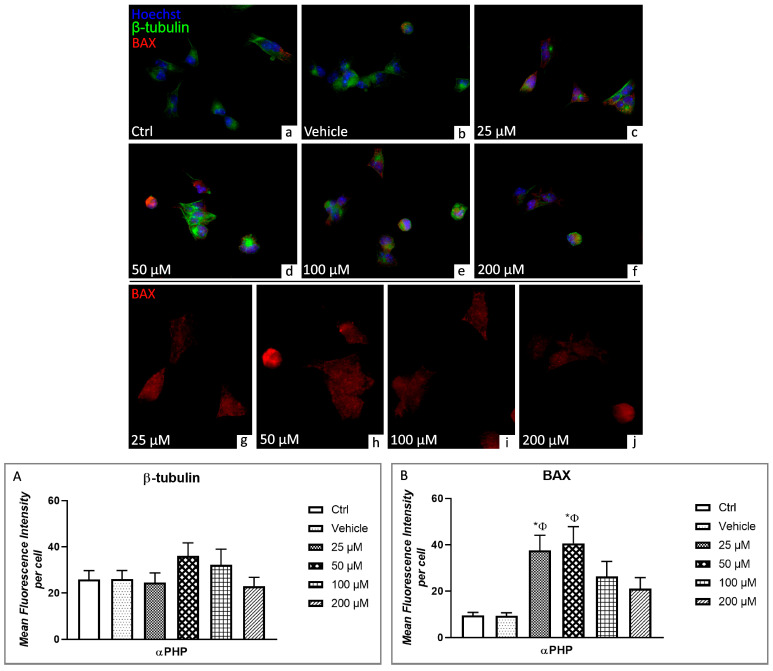
Double immunocytochemical detection of β-tubulin (green signal) and BAX (red signal) by fluorescence microscopy in control (**a**), vehicle (**b**) and differently αPHP-exposed NSPCs, i.e., 25 µM (**c**,**g**), 50 µM (**d**,**h**), 100 µM (**e**,**i**) and 200 µM (**f**,**j**). DNA counterstaining with Hoechst 33258 (blue fluorescence). Histograms depict the quantitative measurement of β-tubulin (**A**) and BAX (**B**) mean fluorescence intensity per cell. Statistically significant data: * compared to ctrl; Φ compared to vehicle. Magnification: 40× (**a**–**f**); 60× (**g**–**j**).

BAX is a member of the Bcl-2 family, able to cross the mitochondrial outer membrane, and key regulator of the intrinsic pathway of apoptosis [65,66,67]. Currently, the double immunofluorescent reaction for BAX (red fluorescence) and β-tubulin (green fluorescence) demonstrated a slight co-localization only in few NSPCs, assessing control and vehicle conditions (Figure 9). Notably, an increase of BAX immunopositivity, typically distinguished by red spots, was detectable already at 25 µM αPHP, further enhancing at 50 µM, evidencing a widespread colocalization of the two molecules, recognizable by the presence of yellow fluorescence. At the highest doses, i.e., 100 and 200 µM αPHP, the yellow fluorescence decreased, as a result of the lessening in the expression levels of the two markers. Nonetheless, the colocalization of BAX and β-tubulin persisted evident even in NSPCs exposed to 200 µM αPHP (Figure 9).

Any significant difference was revealed between control and vehicle cells when measuring BAX immunopositive cell OD. Differently, a significant increase was determined in NSPCs exposed to 25 µM αPHP. This BAX immunopositive cell OD enhancement still remained extremely significant even at 50 µM αPHP. A slight decrease was instead highlighted at 100 µM αPHP, further exacerbated in NSPCs exposed to 200 µM compared to both 25 and 50 µM αPHP. Nonetheless, these BAX immunopositive cell OD levels, measured at the highest doses (100 and 200 µM αPHP) persisted faintly higher compared to those determined in control and vehicle cells (Figure 9, Table S9).

The autophagy protein microtubule-associated protein 1 light chain-3B (LC3B) was assessed based on its crucial regulatory role in the autophagic-apoptosis pathways [68,69,70]. Therefore, a double immunofluorescence reaction was performed to investigate the expression/localization of LC3B (green fluorescence) and lysosomes (red fluorescence) (Figure 6). A complete lack of co-localization between green and red signals was observed in control and vehicle NSPCs (Figure 6). Interestingly, after exposure to 25 µM and 50 µM αPHP, an evident increase of spotted-like red immunofluorescence was detected, in the entire cytoplasm. Remarkably, a dramatic increase of spotted-like green immunofluorescent signal, representative of LC3B, was determined in NSPCs treated with 50 µM αPHP, evident both at nuclear and cytoplasmic level. Notably, these green spots partially co-localize with lysosomes red signals in several NSPCs. The partial colocalization of the two markers even persisted after exposure to the highest αPHP concentration, i.e., 100 and 200 µM, even though a dose-dependent lessening of expression levels was observable (Figure 6).

The subsequent quantitative analyses revealed similar LC3B immunopositive cell OD values comparing control, vehicle cells and NSPCs exposed to 25 µM αPHP. Interestingly, after 50 µM, 100 µM and 200 µM αPHP-exposure, a significant increase of LC3B immunopositive cell OD was detected, compared to control, vehicle and 25 µM αPHP. However, a slight decrease of immunopositive cell OD was measured in NSPCs exposed to 100 µm αPHP compared to 50 µM αPHP. A further slight immunolabeling reduction was observed comparing 200 µm αPHP and 100 µM αPHP (Figure 6, Table S6).

Concerning p62, this versatile protein finely regulates the delicate balance between survival and cell death, being critically involved in several diseases, including CNS injury and neurodegenerative disorders [71,72,73,74].

The double immunofluorescence reaction aimed at determining the expression/localization of p62 (green fluorescence) and Lysosomes (red fluorescence) revealed the lack of co-localization of the two markers (green and red signals, respectively) in control, vehicle cells and NSPCs treated with 25 µm αPHP (Figure 7). However, an evident increase of p62 immunopositivity was observed after exposure to 50 µm αPHP, as clearly demonstrated by the presence of an enhanced green fluorescence signal; notably, a slight colocalization of p62 green signal and lysosomes red signal was disclosed (Figure 7). The increase of p62 immunofluorescence became even more marked in 100 µM αPHP-treated NSPCs, characterized by the presence of several cytoplasmic green spots (Figure 7), but, interestingly, reduced at the highest dose of 200 µM. Remarkably, a complete lack of co-localization between p62 and lysosome fluorescent signals was observed at the highest αPHP doses (Figure 7).

In regard to the quantitative analyses, any difference in p62 immunopositive cell OD was measured comparing control, vehicle cells and NSPCs treated with 25 µM αPHP. Conversely, a very significant increase of p62 immunopositive cell OD was determined in NSPCs treated with 50 µM αPHP, becoming even more significant after exposure to 100 µM αPHP. Interestingly, an extremely significant p62 immunopositive cell OD reduction was revealed at the highest dose (i.e., 200 µM) compared to the lower concentration (100 µM αPHP). As a consequence, any significant difference subsisted between p62 OD values measured in NSPCs treated with 50 µM or 200 µM αPHP, although immunofluorescence persisted significantly higher compared to those determined in control, vehicle and 25 µM αPHP-treated cells (Figure 7, Table S7).

**Figure 10:**
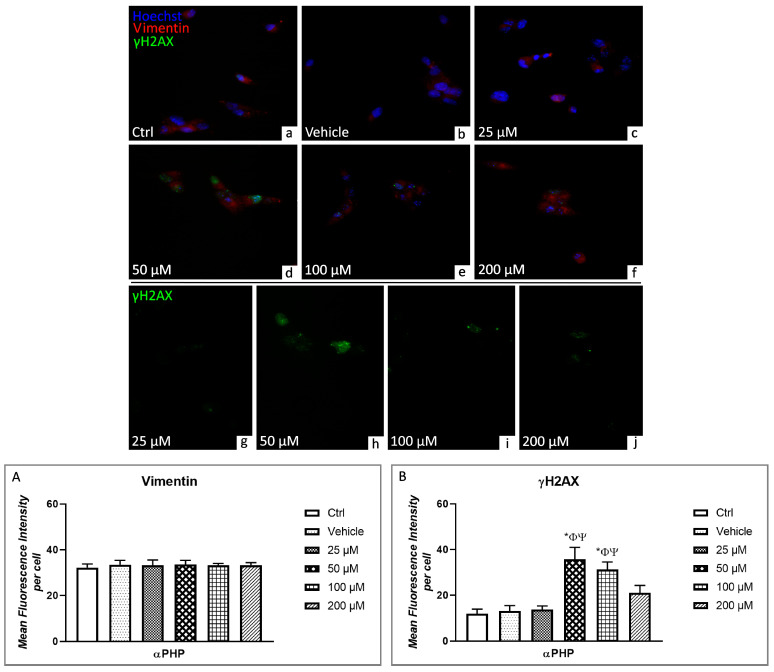
Double immunocytochemical detection of vimentin (red signal) and γH2AX (green signal) by fluorescence microscopy in control (**a**), vehicle (**b**) and differently αPHP-exposed NSPCs, i.e., 25 µM (**c**,**g**), 50 µM (**d**,**h**), 100 µM (**e**,**i**) and 200 µM (**f**,**j**). DNA counterstaining with Hoechst 33258 (blue fluorescence). Histograms depict the quantitative measurement of vimentin (**A**) and γH2AX (**B**) mean fluorescence intensity per cell. Statistically significant data: * compared to ctrl; Φ compared to vehicle; Ψ compared to 25 µM αPHP; # compared to 50 µM αPHP. Magnification: 40× (**a**–**f**); 60× (**g**–**j**).

One of the first molecule involved in DNA damage response is Histone 2AX (H2AX), whose rapid phosphorylation/activation to γH2AX occurred after exposure to DNA-damaging agents to avoid genomic instability [75,76]. The double immunofluorescent reaction for γH2AX (green signal) and Vimentin (red signal) revealed the lack of co-localization of these two markers in all tested experimental condition (Figure 10). A weak green fluorescence was detectable in the nucleoplasm of control, vehicle and 25 µM αPHP exposed cells. Subsequently, after exposure to 50 µM, a significant increase of γH2AX immunopositivity was determined compared to previous groups (control, vehicle and 25 µM), evidently observable in the nucleus as a spotted-like immunofluorescent labelling. However, this green nuclear immunofluorescence tended to decrease after treatment with the higher αPHP concentrations, i.e., 100 µM and 200 µM (Figure 10). In particular, any significant difference in γH2AX immunopositive cell OD was measured comparing control, vehicle and 25 µM αPHP exposed cells. Notably, after exposure to 50 and 100 µM αPHP, a significantly increased immunolabelling was assessed, which nonetheless slightly decreased at 200 µM. However, this latter immunopositivity still remained slightly more marked compared to those assessed in control, vehicle and 25 µM αPHP treated cells (Figure 10). Parallelly, vimentin immunofluorescent signal (red fluorescence), homogeneously distributed throughout the whole cytoplasm, persisted comparable at each evaluated αPHP dose, evidencing the lack of any significative difference among diversely αPHP-exposed NSPCs, showing comparable immunopositive cell OD values (Figure 10, Table S10).

### 3.6. Cell Cycle Distribution and Cell Death

The cytofluorimetric analysis, revealed that, similarly to control, the 100 µM αPHP exposed NPSCs was normally scattered among the different cell phases (G1, S, and G2) showing a physiological DNA content distribution. In detail, only a slight modification in the different cell cycle phases was gauged in αPHP-treated NPSCs, arguably ascribable to biological variability.

**Figure 11:**
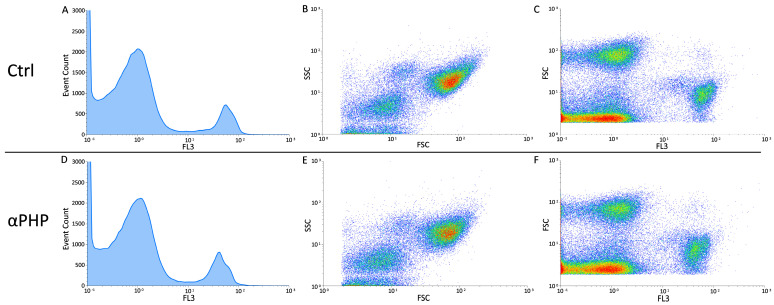
Flow cytometry data reporting live/death NPSCs analysis immediately after αPHP exposure (T0). Cytograms showing the DNA content after propidium iodide (PI) staining in NPSCs ((**A**,**D**), for controls and 100 µM αPHP-treated cells, respectively). Dual parameter cytograms of side scatter (SSC) vs. forward scatter (FSC) and forward scatter (FSC) vs. PI staining (FL3) in controls and αPHP cells ((**B**) vs. (**E**) and (**C**) vs. (**F**), respectively).

**Figure 12:**
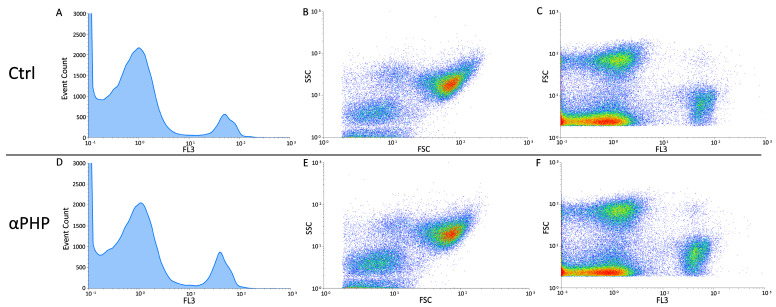
Flow cytometry data reporting live/death NPSCs analysis 15 min after αPHP exposure. Cytograms showing the DNA content after propidium iodide (PI) staining in NPSCs ((**A**,**D**), for controls and 100 µM αPHP treated cells, respectively). Dual parameter cytograms of side scatter (SSC) vs. forward scatter (FSC) and forward scatter (FSC) vs. PI staining (FL3) in controls and αPHP cells ((**B**) vs. (**E**) and (**C**) vs. (**F**), respectively).

**Figure 13:**
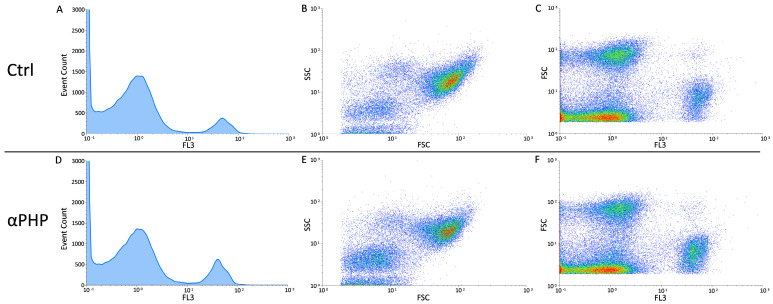
Flow cytometry data reporting live/death NPSCs analysis 30 min after αPHP exposure. Cytograms showing the DNA content after propidium iodide (PI) staining in NPSCs (**A**,**D**), for controls and 100 µM αPHP treated cells, respectively). Dual parameter cytograms of side scatter (SSC) vs. forward scatter (FSC) and forward scatter (FSC) vs. PI staining (FL3) in controls and αPHP cells ((**B**) vs. (**E**) and (**C**) vs. (**F**), respectively).

Differently, concerning the cell death assessment, the biparametric cytograms evaluation revealed that αPHP was able to decrease the number of living cells (about 6%) already after exposure, with a further lessening after 15 min (about 9%) (Figure 11, Figure 12 and Figure 13). Notably, after longer exposure, i.e., 30 min, the reduction was worsened (about 16%) (Figure 11, Figure 12 and Figure 13).

### 3.7. Electrophysiological Recordings

**Figure 14:**
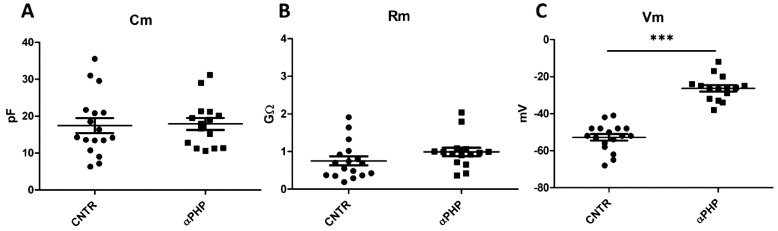
Passive properties of control (*n* = 17) and αPHP treated (*n* = 15) cells. (**A**): Membrane capacitance (Cm, pF); (**B**): Membrane resistance (Rm, GΩ); (**C**): Resting membrane potential (Vm, mV). Statistical results were performed by unpaired *t*-test: *p* < 0.001 (***).

Cells were seeded on glass coverslips (22 mm × 22 mm) located in cell culture dishes (35 mm × 10 mm), were cultured in PM, added with Matrigel, with or without 100 µM αPHP, and were recorded after about 72 h. Control (*n* = 17) and treated (*n* = 15) cells displayed similar passive properties, evaluated as membrane capacitance (Cm = 17.45 ± 2.02 vs. Cm = 17.91 ± 1.62, respectively, Figure 14A) and membrane resistance (Rm = 0.75 ± 0.12 vs. Rm = 0.99 ± 0.11, respectively, Figure 14B).

However, αPHP-treated cells displayed a significant depolarizing resting membrane potential compared to controls (Vm = −23.31 ± 1.72 mV vs. Vm = −52.76 ± 1.78 mV, respectively, Figure 14C).

**Figure 15:**
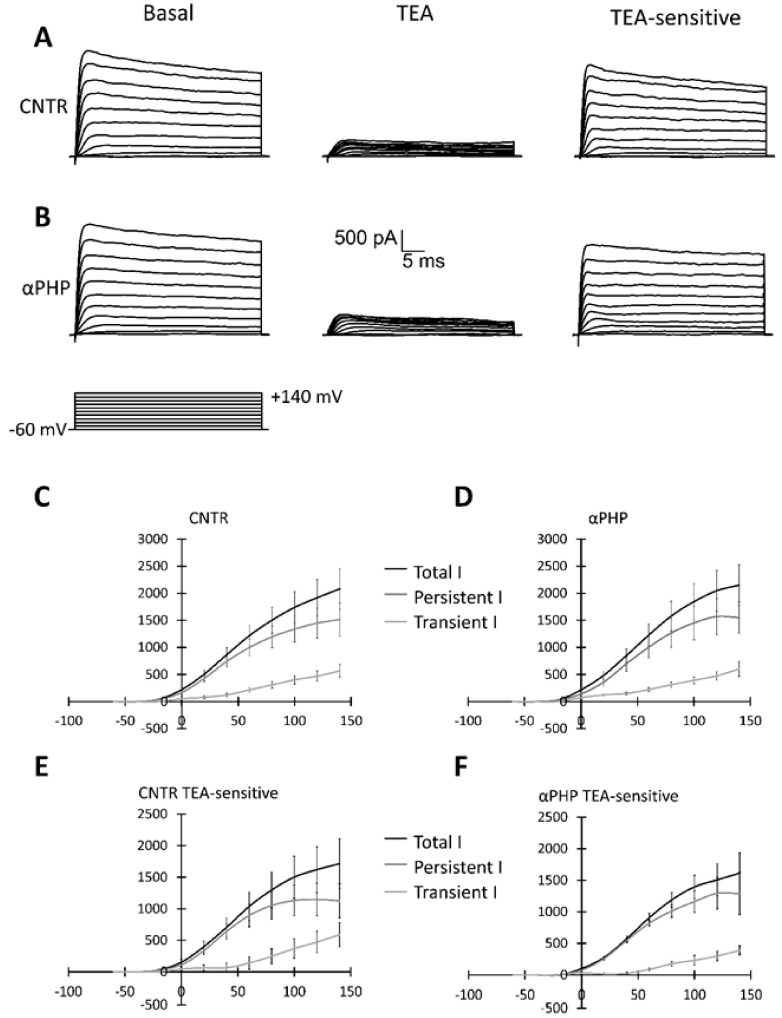
Active electrophysiological properties of control and αPHP treated cells. (**A**): Outward currents recorded in control cells. The currents were blocked by local perfusion with 20 mM TEA (*n* = 10, sampling rate: 20 kHz). (**B**): Outward currents recorded in αPHP treated cells. The currents were blocked by local perfusion with 20 mM TEA (*n* = 6). The currents were evoked using depolarizing voltage steps of +20 mV from a holding potential of −60 mV to +140 mV. The TEA-sensitive currents have been obtained by digital subtraction: basal—TEA. (**C**): Current-voltage relationship for total, transient and persistent currents in control cells (*n* = 17). (**D**): Current-voltage relationship for total, transient and persistent currents in αPHP treated cells (*n* = 15). (**E**): Current-voltage relationship for total, transient and persistent TEA-sensitive currents in control cells (*n* = 10). (**F**): Current-voltage relationship for total, transient and persistent TEA-sensitive currents in αPHP treated cells (*n* = 6).

Regarding active currents, the totality of the control (*n* = 17) and treated (*n* = 15) cells displayed both transient and persistent currents, without any statistical differences. In particular, Figure 15 shows a typical outward current evoked with depolarizing voltage steps of +20 mV from a holding potential of −60 mV to +140 mV, recorded both in control (Figure 15A) and treated (Figure 15B) cells. Notably, the currents were blocked by 20 mM TEA perfusion (Figure 15A,B). Figure 15C,D displays the mean current-voltage relationship for total, transient and persistent currents in control and treated cells, respectively. Figure 15E and F display the mean current-voltage relationship for TEA-sensitive total, transient and persistent currents in control and treated cells, respectively.

### 3.8. Ultrastructural Features by TEM

**Figure 16:**
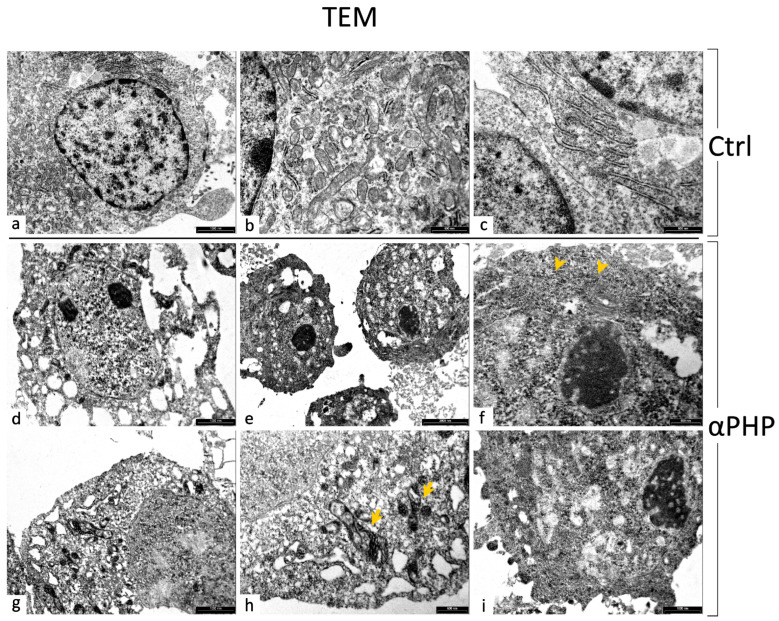
TEM ultrastructural analysis of control (**a**–**c**) and 100 µM αPHP-exposed NSPCs (**d**–**i**). (**d**) Necroptosis. (**e**,**i**) Apoptotic cells. (**g**) Autophagic cell. Arrowheads (**f**) indicate degraded RER while arrows (**h**) show damaged mitochondria after αPHP exposure.

Morphological changes induced by exposure to αPHP were analyzed by electron microscopy. In the control samples, NPSCs displayed the nucleus with physiological decondensed chromatin and showed normal organelles distribution, characterized by the presence of a well-organized rough endoplasmic reticulum (RER) and medium-sized mitochondria, located in the perinuclear area (Figure 16).

Differently, after 100 µM αPHP exposure various ultrastructural alterations were clearly detected. Specifically, NPSCs presented typical changed features related to the activation/progression of different types of cell death pathways. In detail, enlarged nuclei were observed, often characterized by chromatin condensation, absence of the nuclear envelope and partial cytosol degradation. At the cell periphery, the plasma membrane formed some blebs, enclosing cytoplasmic components as a consequence of cytoskeletal damage. Treated NPSCs also showed (i) an elevated number of vacuoles containing cell debris, which could represent distinctive elements of autophagy activation, (ii) a reduced density of mitochondria which appeared severely damaged, and (iii) degraded RER. In addition, several NPCSs exhibited a poorly visible cell membrane (Figure 16).

## 4. Discussion

Nowadays, the overflow NPS phenomenon is taking on public health relevance, alongside and often overlapping with that of traditional drugs. This trend is even more worrying considering the pervasive infiltration of offers on the internet-driven social networks, particularly frequented by adolescents and young adults. In this social setting, the use of SCs is linked to an intensive and repetitive administration pattern in which other drugs are concomitantly consumed, including alcohol [77]. The clinical relevance in cases of NPS toxidromes [1] is also aggravated by the absolute lack of knowledge regarding the medium and long-term effects profile, including their potential for abuse, dependence and withdrawal, up to the possible effects of neurotoxicity that could induce permanent brain deficits. In fact, the related psychiatric problems are relevant.

A crucial link exists between neurogenesis and several psychiatric disorders, but the effects and mechanisms of action of psychoactive substances, including NPS, on neurogenesis still remain unknown or even controversial. Evidence accumulated in the last decade indicating that psychoactive substances negatively influence neurogenesis in the adult, in particular supporting the existence of a mutual relationship between postnatal neurogenesis and addictive behaviors [78,79]. Based on these backgrounds it is reasonable to speculate that SCs could also affect neurogenesis especially in young subjects.

Hence, in the current in vitro study, we investigated the unexplored effect of αPHP on proliferation, survival, cell mechanisms, electrophysiological features, and ultrastructure by using a murine cell-based model, namely NSPCs.

In particular, NSPCs obtained from the SVZ of 8-week-old C57BL/6 mice, were employed to evaluate the toxic effects of αPHP by assessing cell viability/proliferation, clonal capability and selected cellular endpoints, i.e., cell morphology, mitochondrial function, and cell death pathways, also exploring membrane ion channels changes. We revealed that αPHP was able to induce a dose-dependent significant decrease of the viability, proliferation and clonal capability of the NSPCs, paralleled by the resting membrane potential depolarization and apoptotic/autophagic/necroptotic pathway activation. Moreover, ultrastructural alterations were clearly observed.

Concerning the chosen model, it has to be mentioned that, compared to various available cell lines, e.g., SH-SY5Y [80], NT2, and PC12, [81], which allow an easy approach but have to deal with their tumor derivation, neural stem cells have been recognized as effective tools for the study of many different disorders/pathologies including adverse outcomes of drug abuse, taking advantage of their physiological characteristics, which have been intensely studied since their discover [82]. Neural stem cells (NSCs) can be derived from both invertebrates and vertebrates. Concerning the latter, they can be derived from different regions of embryonic and postnatal brain tissue, for instance hippocampus, sub-ventricular zone [34,35] and cerebellum (Bottai, Adami, Rossi, and Canepari unpublished results). The peculiar physiological properties of NSCs consists of their ability to self-maintain, their aptitude to differentiate into the CNS’s cellular subtypes (e.g., neurons, oligodendrocytes, and astrocytes), and the capability to change their functional characteristics in response to the microenvironment.

For clearness, it has to be underlined that mammals brain neurons arise from a small number of neural stem and progenitor cells (NSPCs). NSCs are able to generate all the cell types in the brain, whereas neural progenitors (NPs) have a more restricted potential [36,83]. As a matter of fact, neurogenesis is the process of generation of newborn neurons from NPSCs, located in precise and limited areas, occurring during embryonic and early postnatal stages, but also persisting during the adult mammalian life [38,39], when it plays a fundamental role in guaranteeing neuronal plasticity. Indeed, the CNS is particularly vulnerable to NPS and perturbation of adult neurogenesis may result in several long-lasting, irreversible impairments, including neuroplasticity alteration [31]. Therefore, in the current study we employed NPSCs as a powerful in vitro model [39,40], derived from eight-week-old mice with the goal to mimic the human exposure during puberty, hence trying to translationally assume the potential outcomes of permanent CNS damage in young NPS consumers.

A bulk of literature provides evidence on the neurotoxic mechanisms of SCs highlighting that these NPS can cross the brain-blood barrier, acting like psychostimulants in terms of their effects on neurotransmitter levels in the different brain regions. Intoxication cases and fatalities have been reported in humans, in addition to toxic effects revealed both in lab animals (i.e., rats, mice and zebrafish larvae) as well as in in vitro models. The disclosed toxic effects are often dose- and time-dependent, with different injury extent depending on the diverse tested SC, thus revealing a close relationship between structure and activity [5].

Several in vitro and in vivo studies have been undertaken regarding the toxic SCs outcomes, particularly related to mephedrone, methylone, MDPV and α-PVP exposure, while the effects of αPHP are still scarcely explored. Therefore, we paved the way to fill the gap in the knowledge of αPHP toxicity, by using NSPCs as a useful approach to test SCs.

Accordingly, a recent study testing SCs, demonstrated a significant Mephedrone-induced decrease in the proliferation of NSPCs, cell cycle arrest, paralleled by the enhancement apoptotic and necrotic cells percentages; mephedrone (20–640 µM), also triggered a significant reduction in the number and diameter of neurosphere-forming cells was determined [40].

Our present findings demonstrate for the first time to our knowledge that αPHP induces toxicity in NSPCs of mouse origin, characterized by cell proliferation lessening and apoptosis/autophagy, morphological and ultrastructural alterations, and resting membrane potential depolarization. Notably, the main cytotoxic effects induced by 72 h-exposure to increasing αPHP doses (25–2000 μM) occurred in a concentration-dependent manner.

In particular, the morphological evaluation by phase contrast microscopy, together with viability/proliferation data obtained by proliferation and MTT assays revealed that αPHP caused a dose-dependent cytotoxicity, showing a significant marked effect already at 100 µM, which became more pronounced at the higher doses, i.e., 200 and 500 µM, with the worst damages assessed at the highest concentration, i.e., 1000 and 2000. In particular, the αPHP concentration of 100 µM was able to induce a mild cell injury, causing a decrease of about 35% in the number of living proliferating cells. In addition, the clonogenic assay results revealed that long-lasting (8 days) exposure to increasing αPHP concentrations induced a dose-dependent NSCs colony formation inhibition, with a significant decrease already after exposure to the 100 µM dose and a further clonogenic reduction at 500 μM αPHP, with the highest tested doses, i.e., 1000 and 2000 μM αPHP, able to completely hinder the NSCs clonal capability.

Furthermore, concerning the immunocytochemically evaluated markers, we demonstrated that αPHP was able to dose-dependently affect cell death pathways, altering the expression levels of different proteins crucially involved in these processes, i.e., caspase3, BAX, AIF, LC3B and p62, which showed different dose responses to NPS exposure.

In particular, a significant increase in caspase3 expression levels was determined already at a dose of 100 µM αPHP. At the higher dose of 200 µM, a further enhancement was gauged, notably accompanied by the reduction of mitochondrial expression level (already significant at 100 µM), characterized by the overlapping of the two fluorescence signals, therefore suggesting the contribution of caspase3 in damaging mitochondrial function, through outer membrane permeabilization, and consequent increase in ROS species production, as typical events occurring during apoptosis [84]. Concerning the lessening in mitochondria expression levels, the above reported immunofluorescence data fully matched with the observed ultrastructural alterations characterized by extensive mitochondrial damage accompanied by an evident reduction in their number. Further, a cytoskeletal alteration was disclosed already after exposure to 50 µM αPHP, characterized by changes in β-tubulin expression level, followed by a slight decrease at the highest tested αPHP dose, which could represent a profound cytoskeletal rearrangement depending on caspase activation timing [85] or even the sign of a metabolic shift. In particular, this latter could be a sign of fate change, most likely a differentiation/maturation process under particular conditions, or even an aging-like consequence, especially on mature neurons, indicating that NPS consumption could exert neuronal damaging effect similar to those induced by aging [41,86] in which mitochondrial failure plays a significant role.

Based on these data, we could assume that the mitochondrial depolarization as the result of the rapid increase in active caspase3 levels and the significant cytoskeletal shrinkage represent manifest indicators of apoptotic phenomena occurrence. In support of this hypothesis are our current findings related to BAX and AIF, being key regulator of the apoptotic pathway. In detail, we presently demonstrated that αPHP boosted BAX expression levels already at the lowest tested doses, i.e., 25 and 50 µM, with a diminishing expression level trend at the higher concentrations. In a similar manner, AIF was significantly augmented by αPHP at the concentration of 100 µM, moving from the mitochondria to the cytoplasm, accompanied by tubulin cytoskeletal alterations, then showing a decreasing trend at the higher doses.

Our data are in line with previous studies reporting that mitochondrial outer-membrane permeabilization, by pro-apoptotic Bcl-2 family members, plays a crucial role in apoptosis induction, causing the release of AIF, which seems to define a ‘caspase-dependent’ mitochondria-initiated apoptotic DNA degradation pathway. Further in the intricate balance existing among cell death pathways BAX-mediated mitochondrial release of AIF could be a critical player in early steps of another type of death mechanism, i.e., necroptosis [87], also shown by TEM ultrastructural observation.

Then, it has to be mentioned that the mitochondrial integrity loss could lead to the formation of autophagic compartments and vacuoles. The formation of these structures is an indicator of autophagy, evidencing the initiation of a mitochondrial-mediated autophagy process [88]. In line with this mechanism, in the current investigation we revealed αPHP-induced dose-dependent changes in the expression levels of LC3B and p62, investigated as valuable markers of autophagy, in accordance with previous findings concerning SCs-induced autophagic phenomena [15,71,89,90,91]. Both molecules showed increased expression levels already after exposure to 50 µM αPHP, further enhancing at 100 µM. Notably, the observed LC3B dot-like immunofluorescence, clearly depicted the presence of autophagic vesicles, displayed a partial colocalization with lysosomes, which would be ascribable to the formation of auto-phagolysosomes and autophagosomes [68,70,92], as also supported by ultrastructural evaluations. Therefore, our results on LC3B and p62 expression levels strongly support the activation of the autophagic pathway, in which p62 may play a role. Notably, concerning p62, being an autophagy receptor, its ability to interact with ubiquitinated cargo and LC3B is well known. Date from the previous literature demonstrated that autophagy and p62 are two interdependent parts of the protein control system, strictly interacting to maintain proteostasis, disclosing a frequent p62 upregulation and/or reduced degradation [56].

Our findings are in accordance with several in vitro data, showing that SCs provoke cellular stress and elicit mitochondrial dysfunction, leading to apoptosis and cell deaths consistent with autophagy, and neurodegeneration [15,29,44,45,91]. In accordance, Zhou and their colleagues described that six different SCs, tested in the range of 50–2000 µM, impaired cell membrane integrity, depleted ATP levels, increased mitochondrial superoxide concentrations, and triggered deathly mechanisms, in a concentration-dependent manner. In general, a structure-toxicity profile and a concentration-dependent toxicity were established, with evident differences among the tested compounds, employed models and investigated pathway [5,47,50,93].

Therefore, in line with abundant literature, clearly indicating the existence of an intricate control of cell survival/death modulated by oxidative stress, apoptosis and autophagy in SCs-induced injury, our present data support the occurrence of a complex interplay among deathly mechanisms, i.e., apoptosis/autophagy/necroptosis, involved in αPHP-caused toxicity.

As regards to the potential αPHP capability to alter DNA and cell cycle, our flow cytometry data indicated that 100 µM αPHP unaffected DNA content distribution in exposed NPSCs, displaying a physiological distribution among the different cell phases (G1, S, and G2). About this data, it has nonetheless to be mentioned that, even though apoptosis can be clearly detected, flow cytometry technique cannot distinctly discriminate other form of cell death, i.e., necroptosis [94], whose occurrence was However, demonstrated by current TEM analysis. Hence, it follows that the measured NPSCs population, showing physiological scattering among the different cell phases, could be a “surviving” cells fraction particularly resistant to αPHP treatment.

Moreover, in regard to the hypothesized NPS genotoxic activity, we interestingly revealed that αPHP increased γH2AX expression levels in NPSCs already at a dose of 50 µM, with a further increase at 100 µM. Since the detection of this phosphorylated histone variant has emerged as a highly specific and sensitive molecular marker for monitoring DNA double-strand damage, this finding suggested that αPHP is able to produce a significant genotoxic insult in NPSCs [95,96]. This assumption fits well with a recent in vitro investigation exploring SCs toxicity on TK6 cells, reporting that αPHP neither induced cytotoxic or cytostatic effects nor triggered apoptosis up 100 μM, but, nonetheless significantly increased micronucleus frequency [17].

Overall, the expression levels of all evaluated immunofluorescent markers tended to decrease at the αPHP highest doses, probably due to the massive αPHP-induced cell death/damage/degeneration, which in turn could trigger a complete alteration of physiological cell maintenance mechanisms and related molecules.

Lastly, the electrophysiology experiments confirmed the membrane passive and active biophysical properties (resistance, capacitance, resting membrane potential, and outward currents) of untreated NSPCs already reported in the literature [97,98,99]. In particular, it should be noted that untreated NSPCs membrane potential is settled at about −53 mV. Interestingly, we demonstrated that αPHP was effective to alter the resting membrane potential causing a significant depolarization (from about −53 mV to −23 mV). The genesis, the maintenance, and the regulation of the resting plasma membrane potential is due primarily to the ubiquitous transmembrane protein Na^+^/K^+^-ATPase. Interestingly, apoptotic events [100,101], such as rotenone, a mitochondrial toxin [102], triggers mitochondrial depolarization that is always associated with plasma membrane depolarization through impairment of Na^+^/K^+^-ATPase [102,103]. It was suggested that the mechanism involved in the mitochondrial membrane potential depolarization could be the opening of the permeability transition pore (PTP). The binding of BAX with adenine nucleotide translocator (ANT) is responsible for the PTP formation and triggers the depolarization in the mitochondrial membrane. The depolarization per se increases, in a vicious cycle, releasing apoptogenic factors, such as cytochrome c, in the apoptotic cascade [101].

Accordingly, the presence of broad-spectrum caspase inhibitor [103] or, more specifically, the presence of apoptotic blocker of Bcl2 pathway [102] block the mitochondrial-Na^+^/K^+^-ATPase-depolarization axis. On the other hand, Na^+^/K^+^-ATPase suppression by ouabain significantly enhanced mitochondrial toxin-induced cell apoptosis [102]. In agreement with the suggested link between the apoptotic events and resting membrane potential depolarization, it has been suggested that the hyperpolarization phenotype is one of the mechanisms by which Bcl-2 induces apoptosis resistance [100].

Therefore, the resting membrane potential of exposed NSPCs could be due to a deep mitochondrial damage, as suggested by TEM ultrastructural analysis and by Caspase 3, BAX, and AIF immunocytochemistry data. We suggest that αPHP could be considered a mitochondrial toxin.

## 5. Conclusions

Taken together, our current findings demonstrated for the first time to our knowledge that αPHP provokes toxicity in murine NSPCs, causing cell viability loss, apoptosis/autophagy/necroptosis activation, morphological and ultrastructural alterations, and resting membrane potential depolarization. These concentration-dependent cytotoxic effects were measured after 72 h-exposure to increasing αPHP doses (25–2000 μM). Our data demonstrated that αPHP, which are able to damage NPCSs, the key players in the neurogenic niche, may affect adult neurogenesis, possibly triggering long-lasting, irreversible CNS damages. As a needed continuation, our ongoing studies are still assessing whether and how this SC affects differentiated cells, exploring if different effects occurred on diverse cellular subtypes, e.g., neurons, oligodendrocytes, and astrocytes, with the final aim of contributing to broadening the knowledge of SCs toxicology, which is necessary to establish an appropriate treatment for NPS and the potential consequences for public health.

## Figures and Tables

**Table 1:** Primary and secondary antibodies employed for experimental procedures.

	Antigen	Immunogen	Manufacturer, Species, Mono-Polyclonal, Cat./Lot. No., RRID	Dilution
Primary antibodies	Anti-β-tubulin (H-235):	Purified antibody raised against amino acids 210–444 mapping at the C-terminus of β-Tubulin of human origin.	Santa Cruz Biotechnology (Santa Cruz, CA, USA), Rabbit polyclonal IgG, Cat# sc-9104, RRID: AB_2241191	1:200
	Anti-apoptosis-inducing factor (E-1)	Purified antibody raised against amino acids 1–300 of AIF of human origin	Santa Cruz Biotechnology (Santa Cruz, CA, USA), Mouse monoclonal IgG, Cat# sc-13116, RRID: AB_626654	1:100
	Anti-Bcl-2-associated X protein (P-19)	Purified antibody raised against a peptide mapping at the N-terminus of BAX of mouse origin	Santa Cruz Biotechnology (Santa Cruz, CA, USA), Rabbit polyclonal IgG, Cat# sc-526, RRID: AB_2064668	1:100
	Anti- caspase-3 (31A1067)	Purified antibody raised against amino acids 50–86 of caspase-3 of human origin	Santa Cruz Biotechnology (Santa Cruz, CA, USA), Mouse monoclonal IgG, Cat# sc-56053, RRID: AB_781826	1:100
	Anti-Histone H2A.X (6L16)	Purified antibody raised against KLH-conjugated linear peptide corresponding to 9 amino acids surrounding serine 139 of human Histone H2AX	Merck KGaA (Darmstadt, Germany), Rabbit Monoclonal Antibody, Cat# ZRB05636, RRID: AB_309864.	1:100
	Anti-p62/SQSTM1	Recombinant full-length protein corresponding to Human SQSTM1/p62 aa 1–440	Abcam (Cambridge, United States), Mouse monoclonal, Cat# ab56416, RRID: AB_945626	1:100
	Anti-Mitochondria serum	Human autoimmune serum recognizing the 70 kDa E2 subunit of pyruvate dehydrogenase complex (kindly given by IRCCS San Matteo Pavia, Italy) ^a^		1:300
	Anti-LC3B	Rabbit polyclonal anti-LC3B (Cell Signaling Technology, Danvers, United States)	Cell Signaling Technology, (Danvers, United States), Rabbit polyclonal, Cat# 2775S, RRID: AB_915950	1:200
	Anti-Lysosomes serum	Human autoimmune serum (kindly given by IRCCS San Matteo Pavia, Italy) ^b^		1:500
	Anti-Vimentin (V-9)	Purified vimentin from pig eye lens	Thermo Fisher Scientific (Monza, Italy), Mouse monoclonal, Cat# MA5-11883, RRID: AB_10985392	1:200
Secondary antibodies	Alexa Fluor™ 488 goat anti-rabbit IgG (H + L) Highly Cross-Adsorbed Secondary Antibody	Gamma Immunoglobins Heavy and Light chains	Thermo Fisher Scientific (Monza, Italy)	1:200
	Alexa Fluor™ 488 goat anti-mouse IgG (H + L) Highly Cross-Adsorbed Secondary Antibody	Gamma Immunoglobins Heavy and Light chains	Thermo Fisher Scientific (Monza, Italy)	1:200
	Alexa Fluor™ 594 goat anti-rabbit IgG (H + L) Highly Cross-Adsorbed Secondary Antibody	Gamma Immunoglobins Heavy and Light chains	Thermo Fisher Scientific (Monza, Italy)	1:200
	Alexa Fluor™ 594 goat anti-mouse IgG (H + L) Highly Cross-Adsorbed Secondary Antibody	Gamma Immunoglobins Heavy and Light chains	Thermo Fisher Scientific (Monza, Italy)	1:200
	Alexa Fluor™ 594 goat anti-human IgG (H + L) Highly Cross-Adsorbed Secondary Antibody	Gamma Immunoglobins Heavy and Light chains	Thermo Fisher Scientific (Monza, Italy)	1:200
	Alexa Fluor™ 647 goat anti-human IgG (H + L) Highly Cross-Adsorbed Secondary Antibody	Gamma Immunoglobins Heavy and Light chains	Thermo Fisher Scientific (Monza, Italy)	1:200

^a^ [56]; ^b^ [57].

## Data Availability

The datasets used and analyzed during the current investigation are available from the corresponding author upon equitable request.

## Supplementary Materials

The following supporting information can be downloaded at: https://www.mdpi.com/article/10.3390/biology12091225/s1, Figure S1. Immunocytochemical detection of different specific marker of undifferentiated CNS cells: Nestin (red signal in a), O4 (red signal in b), SOX2 (green signal in c), β-tubulin III (red signal in d and f) and GFAP (green signal in e and f) by fluorescence microscopy in NSPCs. DNA counterstaining with Hoechst 33258 (blue fluorescence). Magnification: 20× (a); 40× (b,c); 60× (d–f). Table S1. Statistical analyses of cellular proliferation (%). One-way ANOVA (Bonferroni multiple comparisons test) (Figure 1). ns: not significative; *p* < 0.01 (**); *p* < 0.001 (***). Table S2. Statistical analyses of cell density using Phase-contrast microscopy. One-way ANOVA (Bonferroni multiple comparisons test) (Figure 2). ns: not significative; *p* < 0.001 (***). Table S3. Statistical analyses of MTT assay. One-way ANOVA (Bonferroni multiple comparisons test) (Figure 3). ns: not significative; *p* < 0.05 (*); *p* < 0.01 (**); *p* < 0.001 (***). Table S4. Statistical analyses of survival fraction (%). One-way ANOVA (Bonferroni multiple comparisons test) (Figure 4). ns: not significative; *p* < 0.001 (***). Table S5. Statistical analyses of β-tubulin, mitochondria and caspase 3 mean fluorescence intensity per cell evaluated using confocal microscopy (Figure 5). Table S6. Statistical analyses of Lysosomes and LC3B mean fluorescence intensity per cell (Figure 6). Table S7. Statistical analyses of Lysosomes and p62 mean fluorescence intensity per cell (Figure 7). Table S8. Statistical analyses of β-tubulin and AIF mean fluorescence intensity per cell (Figure 8). Table S9. Statistical analyses of β-tubulin and BAX mean fluorescence intensity per cell (Figure 9). Table S10. Statistical analyses of Vimentin and yH2AX mean fluorescence intensity per cell (Figure 10).
