# Supplementary material for: The Designer Drug αPHP Affected Cell Proliferation and Triggered Deathly Mechanisms in Murine Neural Stem/Progenitor Cells
Source: Biology (Basel). 2023 Sep 11;12(9):1225. doi: 10.3390/biology12091225 (PMC10525791; doi:10.3390/biology12091225)
Supplement: Supplementary file 1 [file biology-12-01225-s001.zip › biology-2508638-supplementary.pdf]

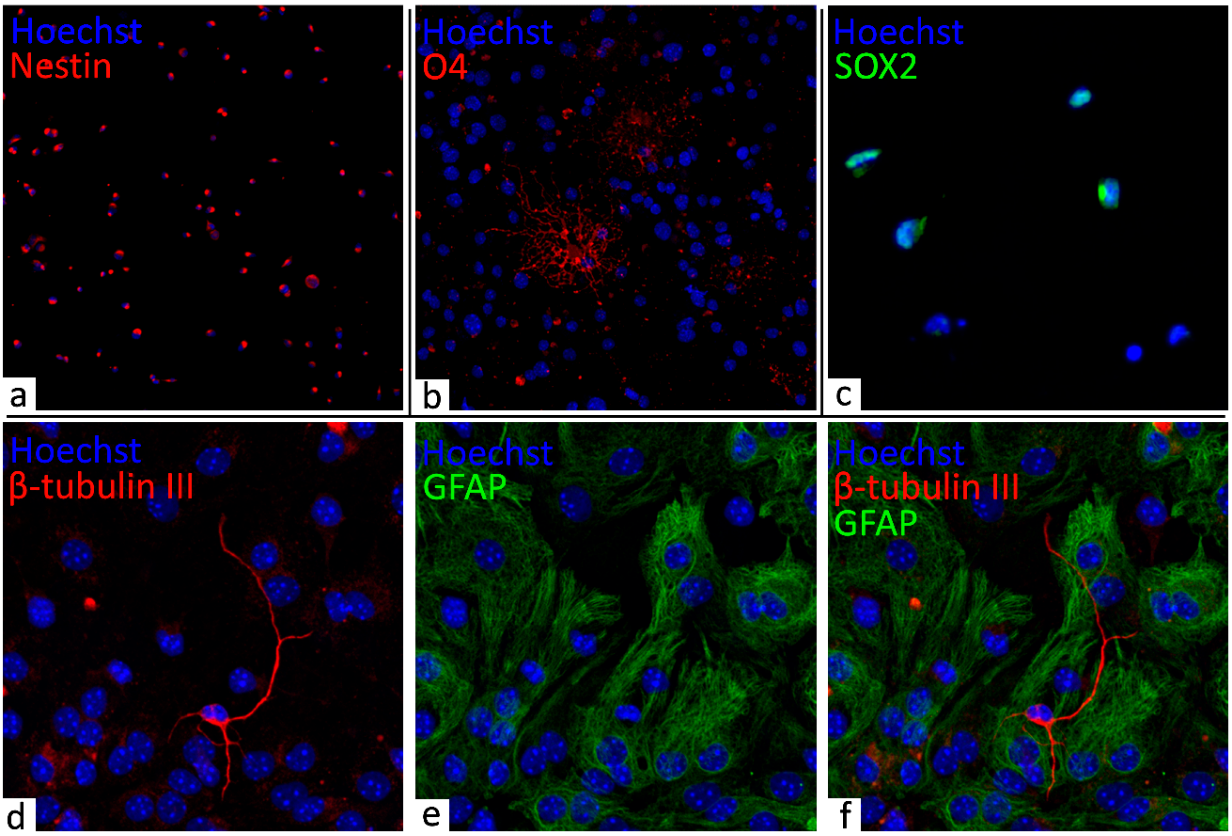

Figure S1. Immunocytochemical detection of different specific marker of undifferentiated CNS cells: Nestin (red signal in a), O4 (red signal in b), SOX2 (green signal in c), β-tubulin III (red signal in d and f) and GFAP (green signal in e and f) by fluorescence microscopy in NSPCs. DNA counterstaining with Hoechst 33258 (blue fluorescence). Magnification: 20× (a); 40× (b-c); 60× (d-f).

**Cell number (%)**	Ctrl	Vehicle	Ethanol	25 µM	50 µM	100 µM	200 µM	500 µM	1000 µM	2000 µM
Ctrl (100.00±0.00)	---	*ns*	***	**	***	***	***	***	***	***
Vehicle (100.00±0.00)	*ns*	---	***	**	***	***	***	***	***	***
Ethanol (27.82±2.20)	***	***	---	***	***	**	*ns*	*ns*	**	**
25 µM (84.27±4.97)	**	**	***	---	***	***	***	***	***	***
50 µM (66.24±4.17)	***	***	***	***	---	*ns*	***	***	***	***
100 µM (53.72±4.17)	***	***	**	***	*ns*	---	***	***	***	***
200 µM (30.68±3.78)	***	***	*ns*	***	***	***	---	***	***	***
500 µM (6.59±1.20)	***	***	*ns*	***	***	***	***	---	*ns*	*ns*
1000 µM (0.00±0.00)	***	***	**	***	***	***	***	*ns*	---	*ns*
2000 µM (0.00±0.00)	***	***	**	***	***	***	***	*ns*	*ns*	---

Table S1. Statistical analyses of cellular proliferation (%). One-way ANOVA (Bonferroni multiple comparisons test) (Figure 1). ns: not significative; $p<0.01$ (**); $p<0.001$ (***).

**Cell density**	Ctrl	Vehicle	25 μM	50 μM	100 μM	200 μM	500 μM	1000 μM	2000 μM
Ctrl (110.56±1.83)	---	*ns*	***	***	***	***	***	***	***
Vehicle (110.84±2.21)	*ns*	---	***	***	***	***	***	***	***
25 μM (84.24±1.88)	***	***	---	*ns*	***	***	***	***	***
50 μM (78.97±1.65)	***	***	*ns*	---	***	***	***	***	***
100 μM (57.91±1.00)	***	***	***	***	---	***	***	***	***
200 μM (47.38±0.55)	***	***	***	***	***	---	***	***	***
500 μM (16.58±0.21)	***	***	***	***	***	***	---	***	***
1000 μM (4.69±0.03)	***	***	***	***	***	***	***	---	*ns*
2000 μM (0.00±0.00)	***	***	***	***	***	***	***	*ns*	---

Table S2. Statistical analyses of cell density using Phase-contrast microscopy. One-way ANOVA (Bonferroni multiple comparisons test) (Figure 2). ns: not significative; $p<0.001$ (***).

Cell viability	Ctrl	Vehicle	Ethanol	25 μM	50 μM	100 μM	200 μM	500 μM	1000 μM	2000 μM
Ctrl (1.16±0.19)	---	*ns*	***	*	**	***	***	***	***	***
Vehicle (1.14±0.16)	*ns*	---	***	*	*	***	***	***	***	***
Ethanol (0.12±0.00)	***	***	---	***	***	***	***	***	*	*ns*
25 μM (0.87±0.13)	*	*	***	---	*ns*	*ns*	*ns*	*ns*	***	***
50 μM (0.83±0.11)	**	*	***	*ns*	---	*ns*	*ns*	*ns*	***	***
100 μM (0.76±0.09)	***	***	***	*ns*	*ns*	---	*ns*	*ns*	**	***
200 μM (0.75±0.11)	***	***	***	*ns*	*ns*	*ns*	---	*ns*	**	***
500 μM (0.68±0.14)	***	***	***	*ns*	*ns*	*ns*	*ns*	---	*ns*	***
1000 μM (0.44±0.08)	***	***	*	***	***	**	**	*ns*	---	***
2000 μM (0.06±0.03)	***	***	*ns*	***	***	***	***	***	***	---

Table S3. Statistical analyses of MTT assay. One-way ANOVA (Bonferroni multiple comparisons test) (Figure 3). ns: not significative; $p<0.05$ (*); $p<0.01$ (**); $p<0.001$ (***).

SF (%)	Ctrl	Vehicle	25 µM	50 µM	100 µM	200 µM	500 µM	1000 µM	2000 µM
Ctrl (100.00±0.00)	---	*ns*	*ns*	*ns*	***	***	***	***	***
Vehicle (100.00±0.00)	*ns*	---	*ns*	*ns*	***	***	***	***	***
25 µM (91.75±4.65)	*ns*	*ns*	---	*ns*	*ns*	*ns*	***	***	***
50 µM (88.80±5.63)	*ns*	*ns*	*ns*	---	*ns*	*ns*	***	***	***
100 µM (77.21±5.69)	***	***	*ns*	*ns*	---	*ns*	***	***	***
200 µM (78.79±4.72)	***	***	*ns*	*ns*	*ns*	---	***	***	***
500 µM (34.43±5.06)	***	***	***	***	***	***	---	***	***
1000 µM (0.00±0.00)	***	***	***	***	***	***	***	---	*ns*
2000 µM (0.00±0.00)	***	***	***	***	***	***	***	*ns*	---

Table S4. Statistical analyses of survival fraction (%). One-way ANOVA (Bonferroni multiple comparisons test) (Figure 4). ns: not significative; p<0.001 (***).

**β-tubulin**	Ctrl (13.79±2.05)	Vehicle (13.97±2.02)	25 µM (16.46±3.25)	50 µM (45.89±7.74)	100 µM (32.93±5.26)	200 µM (29.88±2.62)
Ctrl	---	*ns*	*ns*	***	*ns*	*ns*
Vehicle	*ns*	---	*ns*	***	*ns*	*ns*
25 µM	*ns*	*ns*	---	***	*ns*	*ns*
50 µM	***	***	***	---	*ns*	*ns*
100 µM	*ns*	*ns*	*ns*	*ns*	---	*ns*
200 µM	*ns*	*ns*	*ns*	*ns*	*ns*	---

One-way ANOVA (Bonferroni multiple comparisons test). ns: not significative; p<0.001 (***).

**Mitochondria**	Ctrl (36.68±2.98)	Vehicle (37.61±2.17)	25 µM (41.67±2.04)	50 µM (49.83±7.11)	100 µM (22.48±1.92)	200 µM (11.44±2.40)
Ctrl	---	*ns*	*ns*	*ns*	*ns*	***
Vehicle	*ns*	---	*ns*	*ns*	*ns*	***
25 µM	*ns*	*ns*	---	*ns*	***	***
50 µM	*ns*	*ns*	*ns*	---	***	***
100 µM	*ns*	*ns*	***	***	---	*ns*
200 µM	***	***	***	***	*ns*	---

One-way ANOVA (Bonferroni multiple comparisons test). ns: not significative; p<0.001 (***).

**Caspase 3**	Ctrl (0.15±0.10)	Vehicle (0.15±0.08)	25 µM (0.12±0.06)	50 µM (0.79±0.31)	100 µM (2.04±0.33)	200 µM (3.94±0.18)
Ctrl	---	*ns*	*ns*	*ns*	**	***
Vehicle	*ns*	---	*ns*	*ns*	**	***
25 µM	*ns*	*ns*	---	*ns*	*	***
50 µM	*ns*	*ns*	*ns*	---	*ns*	*
100 µM	**	**	*	*ns*	---	*ns*
200 µM	***	***	***	*	*ns*	---

Kruskal-Wallis test (Dunn's multiple comparisons test). ns: not significative; p<0.05 (*); p<0.01 (**); p<0.001 (***).

Table S5. Statistical analyses of β-tubulin, mitochondria and caspase 3 mean fluorescence intensity per cell evaluated using confocal microscopy (Figure 5).

**Lysosomes**	Ctrl (9.33±2.21)	Vehicle (8.38±1.97)	25 μM (25.16±3.45)	50 μM (31.77±18.15)	100 μM (19.20±2.82)	200 μM (16.11±4.25)
Ctrl	---	*ns*	*	***	*ns*	*ns*
Vehicle	*ns*	---	*	***	*ns*	*ns*
25 μM	*	*	---	*ns*	*ns*	*ns*
50 μM	***	***	*ns*	---	*ns*	*ns*
100 μM	*ns*	*ns*	*ns*	*ns*	---	*ns*
200 μM	*ns*	*ns*	*ns*	*ns*	*ns*	---

One-way ANOVA (Bonferroni multiple comparisons test). ns: not significative. $p<0.05$ (*); $p<0.001$ (***).

**LC3B**	Ctrl (0.25±0.06)	Vehicle (0.25±0.05)	25 μM (0.39±0.08)	50 μM (14.16±1.91)	100 μM (11.18±1.45)	200 μM (9.33±0.83)
Ctrl	---	*ns*	*ns*	***	***	***
Vehicle	*ns*	---	*ns*	***	***	***
25 μM	*ns*	*ns*	---	**	**	*
50 μM	***	***	**	---	*ns*	*ns*
100 μM	***	***	**	*ns*	---	*ns*
200 μM	***	***	*	*ns*	*ns*	---

Kruskal-Wallis test (Dunn's multiple comparisons test). ns: not significative; $p<0.05$ (*); $p<0.01$ (**); $p<0.001$ (***).

Table S6. Statistical analyses of Lysosomes and LC3B mean fluorescence intensity per cell (Figure 6).

**Lysosomes**	Ctrl (10.13±2.32)	Vehicle (10.26±2.23)	25 µM (30.58±3.98)	50 µM (33.10±5.77)	100 µM (19.96±3.09)	200 µM (18.04±1.98)
Ctrl	---	*ns*	***	***	*ns*	*ns*
Vehicle	*ns*	---	***	**	*ns*	*ns*
25 µM	***	***	---	*ns*	*ns*	*ns*
50 µM	***	**	*ns*	---	*ns*	*ns*
100 µM	*ns*	*ns*	*ns*	*ns*	---	*ns*
200 µM	*ns*	*ns*	*ns*	*ns*	*ns*	---

Kruskal-Wallis test (Dunn's multiple comparisons test). ns: not significative.

**p62**	Ctrl (0.20±0.04)	Vehicle (0.21±0.05)	25 µM (0.22±0.04)	50 µM (10.58±1.32)	100 µM (22.48±3.65)	200 µM (9.98±1.35)
Ctrl	---	*ns*	*ns*	**	***	**
Vehicle	*ns*	---	*ns*	**	***	**
25 µM	*ns*	*ns*	---	**	***	**
50 µM	**	**	**	---	***	*ns*
100 µM	***	***	***	***	---	***
200 µM	**	**	**	*ns*	***	---

One-way ANOVA (Bonferroni multiple comparisons test). ns: not significative; p<0.01 (**); p<0.001 (***).

Table S7. Statistical analyses of Lysosomes and p62 mean fluorescence intensity per cell (Figure 7).

**β-tubulin**	Ctrl (21.17±4.48)	Vehicle (21.05±4.32)	25 µM (22.14±3.26)	50 µM (22.92±3.90)	100 µM (22.92±4.53)	200 µM (22.07±3.72)
Ctrl	---	*ns*	*ns*	*ns*	*ns*	*ns*
Vehicle	*ns*	---	*ns*	*ns*	*ns*	*ns*
25 µM	*ns*	*ns*	---	*ns*	*ns*	*ns*
50 µM	*ns*	*ns*	*ns*	---	*ns*	*ns*
100 µM	*ns*	*ns*	*ns*	*ns*	---	*ns*
200 µM	*ns*	*ns*	*ns*	*ns*	*ns*	---

Kruskal-Wallis test (Dunn's multiple comparisons test). ns: not significative.

**AIF**	Ctrl (7.12±0.71)	Vehicle (6.87±0.87)	25 µM (7.06±1.65)	50 µM (7.71±1.80)	100 µM (27.39±8.14)	200 µM (16.86±2.84)
Ctrl	---	*ns*	*ns*	*ns*	***	*ns*
Vehicle	*ns*	---	*ns*	*ns*	***	*ns*
25 µM	*ns*	*ns*	---	*ns*	***	*ns*
50 µM	*ns*	*ns*	*ns*	---	***	*ns*
100 µM	***	***	***	***	---	*ns*
200 µM	*ns*	*ns*	*ns*	*ns*	*ns*	---

One-way ANOVA (Bonferroni multiple comparisons test). ns: not significative; $p<0.001$ (***).

Table S8. Statistical analyses of β-tubulin and AIF mean fluorescence intensity per cell (Figure 8).

**β-tubulin**	Ctrl (25.91±3.84)	Vehicle (26.00±3.76)	25 μM (24.53±5.18)	50 μM (36.08±5.65)	100 μM (32.18±6.77)	200 μM (22.95±3.86)
Ctrl	---	*ns*	*ns*	*ns*	*ns*	*ns*
Vehicle	*ns*	---	*ns*	*ns*	*ns*	*ns*
25 μM	*ns*	*ns*	---	*ns*	*ns*	*ns*
50 μM	*ns*	*ns*	*ns*	---	*ns*	*ns*
100 μM	*ns*	*ns*	*ns*	*ns*	---	*ns*
200 μM	*ns*	*ns*	*ns*	*ns*	*ns*	---

Kruskal-Wallis test (Dunn's multiple comparisons test). ns: not significative.

**BAX**	Ctrl (9.57±1.33)	Vehicle (9.39±1.30)	25 μM (37.56±6.54)	50 μM (40.65±7.24)	100 μM (26.33±6.52)	200 μM (21.08±4.80)
Ctrl	---	*ns*	**	***	*ns*	*ns*
Vehicle	*ns*	---	**	***	*ns*	*ns*
25 μM	**	**	---	*ns*	*ns*	*ns*
50 μM	***	***	*ns*	---	*ns*	*ns*
100 μM	*ns*	*ns*	*ns*	*ns*	---	*ns*
200 μM	*ns*	*ns*	*ns*	*ns*	*ns*	---

One-way ANOVA (Bonferroni multiple comparisons test). ns: not significative; p<0.01 (**); p<0.001 (***).

Table S9. Statistical analyses of β-tubulin and BAX mean fluorescence intensity per cell (Figure 9).

**Vimentin**	Ctrl (32.28±1.55)	Vehicle (33.48±1.88)	25 μM (33.29±2.28)	50 μM (33.50±1.85)	100 μM (33.24±0.79)	200 μM (33.34±1.10)
Ctrl	---	*ns*	*ns*	*ns*	*ns*	*ns*
Vehicle	*ns*	---	*ns*	*ns*	*ns*	*ns*
25 μM	*ns*	*ns*	---	*ns*	*ns*	*ns*
50 μM	*ns*	*ns*	*ns*	---	*ns*	*ns*
100 μM	*ns*	*ns*	*ns*	*ns*	---	*ns*
200 μM	*ns*	*ns*	*ns*	*ns*	*ns*	---

Kruskal-Wallis test (Dunn's multiple comparisons test). ns: not significative.

**yH2AX**	Ctrl (11.99±1.92)	Vehicle (13.18±2.37)	25 μM (13.74±1.62)	50 μM (35.84±5.13)	100 μM (31.26±3.29)	200 μM (21.14±3.21)
Ctrl	---	*ns*	*ns*	**	**	*ns*
Vehicle	*ns*	---	*ns*	**	**	*ns*
25 μM	*ns*	*ns*	---	**	**	*ns*
50 μM	**	**	**	---	*ns*	*ns*
100 μM	**	**	**	*ns*	---	*ns*
200 μM	*ns*	*ns*	*ns*	*ns*	*ns*	---

Kruskal-Wallis test (Dunn's multiple comparisons test). ns: not significative; p<0.01 (**).

Table S10. Statistical analyses of Vimentin and yH2AX mean fluorescence intensity per cell (Figure 10).
